# Tissue mechanics promote IDH1-dependent HIF1α–tenascin C feedback to regulate glioblastoma aggression

**DOI:** 10.1038/ncb3429

**Published:** 2016-11-07

**Authors:** Yekaterina A. Miroshnikova, Janna K. Mouw, J. Matthew Barnes, Michael W. Pickup, Johnathan N. Lakins, Youngmi Kim, Khadjia Lobo, Anders I. Persson, Gerald F. Reis, Tracy R. McKnight, Eric C. Holland, Joanna J. Phillips, Valerie M. Weaver

**Affiliations:** 1 Center for Bioengineering and Tissue Regeneration, Department of Surgery, University of California San Francisco, San Francisco, California 94143, USA; 2 Division of Human Biology and Solid Tumor Translational Research, Fred Hutchinson Cancer Research Center, Department of Neurosurgery and Alvord Brain Tumor Center, University of Washington, Seattle, Washington 98109, USA; 3 Magnetic Resonance Science Center, Department of Radiology and Biomedical Imaging, University of California San Francisco, San Francisco, California 94143, USA; 4 Department of Neurology, University of California, San Francisco, California 94143, USA; 5 Department of Neurological Surgery, University of California San Francisco, San Francisco, California 94158, USA; 6 Brain Tumor Research Center, Helen Diller Family Cancer Research Center, University of California San Francisco, San Francisco, California 94143, USA; 7 UCSF Comprehensive Cancer Center, Helen Diller Family Cancer Research Center, University of California San Francisco, San Francisco, California 94143, USA; 8 Department of Pathology, University of California, San Francisco, California 94143, USA; 9 Department of Anatomy and Department of Bioengineering and Therapeutic Sciences, University of California San Francisco, San Francisco, California 94143, USA; 10 Eli and Edythe Broad Center of Regeneration Medicine and Stem Cell Research, University of California, San Francisco, San Francisco, California 94143, USA; 11 UCSF Helen Diller Comprehensive Cancer Center, University of California, San Francisco, San Francisco, California 94143, USA

## Abstract

Increased overall survival for patients with glioma brain tumours is associated with mutations in the metabolic regulator isocitrate dehydrogenase 1 (IDH1). Gliomas develop within a mechanically challenged microenvironment that is characterized by a dense extracellular matrix (ECM) that compromises vascular integrity to induce hypoxia and activate HIF1α. We found that glioma aggression and patient prognosis correlate with HIF1α levels and the stiffness of a tenascin C (TNC)-enriched ECM. Gain- and loss-of-function xenograft manipulations demonstrated that a mutant IDH1 restricts glioma aggression by reducing HIF1α-dependent TNC expression to decrease ECM stiffness and mechanosignalling. Recurrent IDH1-mutant patient gliomas had a stiffer TNC-enriched ECM that our studies attributed to reduced miR-203 suppression of HIF1α and TNC mediated via a tension-dependent positive feedback loop. Thus, our work suggests that elevated ECM stiffness can independently foster glioblastoma aggression and contribute to glioblastoma recurrence via bypassing the protective activity of IDH1 mutational status.

Malignant gliomas are a highly heterogeneous group of brain tumours that exhibit diverse invasiveness, aggressiveness and treatment responsiveness. Diffuse lower-grade gliomas (LGGs, World Health Organization (WHO) grades II and III) demonstrate highly variable clinical behaviour ranging from indolent to rapidly progressive^1^. LGGs often recur as glioblastomas (GBMs, WHO grade IV glioma), which are inarguably the most advanced and lethal adult brain tumour, with poor treatment responsiveness and a high recurrence rate contributing to poor patient outcome.

Mutations in the metabolic enzyme isocitrate dehydrogenase (IDH) characterize the majority of LGGs and a small percentage of GBMs, and define subtypes that associate with better responses to radiation treatment and an improved prognosis compared with gliomas with wild-type (WT) IDH^1–4^. Oncogenic IDH mutations divert metabolic flux resulting in high levels of (*R*)-2-hydroxyglutarate, an onco-metabolite implicated in hypoxia-inducible factor 1 alpha (HIF1α) stability, epigenetic modifications and extracellular matrix (ECM) remodelling^5^. The relationship between IDH mutations and downstream HIF1α signalling remains unclear with conflicting reports suggesting elevated HIF1α protein in IDH-mutant tumours^6^ or, conversely, blunted hypoxia sensing through HIFα mediated by elevated activity of prolyl 4-hydroxylases^7^. As tumours are characterized by altered tissue-level and cellular mechanics, including ECM remodelling and stiffening, and a stiffened ECM can compromise blood vessel integrity to induce hypoxia and activate HIF1α (refs 8–10), we investigated the interplay between IDH1 mutation status, ECM mechanics, HIF1α signalling and GBM aggression.

## Results

### ECM stiffness associates with IDH1 mutations in primary tumours

**Figure 1:**
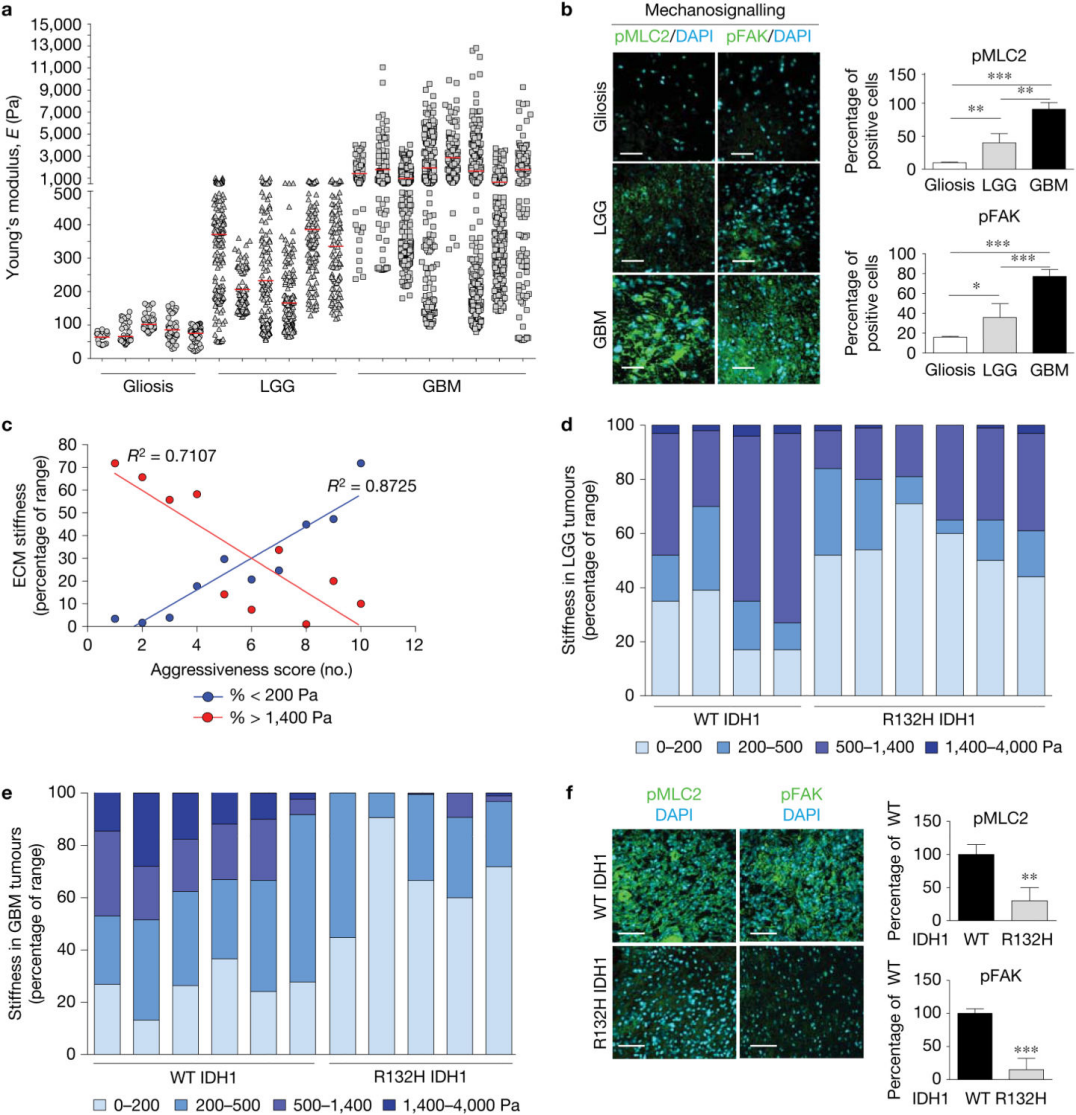
ECM stiffness associates with IDH1 mutations in primary GBM tumours. **(a)** Distribution of ECM stiffness in non-malignant gliotic (Gliosis, *n* = 5 patient samples), WT IDH WHO grades II–III *de novo* (primary) glioma (LGG, *n* = 6 patient samples) and WT IDH WHO grade IV primary GBM (GBM, *n* = 8 patient samples) human patient samples as measured by AFM. Ten regions per patient are shown to illustrate mechanical heterogeneity (pooled patient means indicated with red lines). For statistical analysis, all maps were pooled per patient, and *n* values are patients per group (two-sided Kolmogorov-Smirnov test, *P*=1.1 × 10^−4^ for LGG versus Gliosis and *P* = 3.23×10^−5^ for GBM versus Gliosis). **(b)** Immunofluorescence images and quantification for pMLC2 (green, left) and pFAK397 (green, right) with DAPI (blue) for the tumours shown in **a** (mean ± s.d., *n* = 5 patient samples per group, one-way ANOVA with Tukey's multiple comparisons test, *P<0.05, **P<0.01, ***P< 0.001). **(c)** NanoString nCounter gene expression analysis score (binned on a scale from 1–10 with 1 indicating the most aggressive) plotted against proportion of highly stiff (*E*> 1,400 Pa, red) or soft (*E*<200Pa, blue) ECM areas (*n*=10 patient samples, linear regression where *R*^2^ = 0.7107 for >1,400Pa and *R*^2^ = 0.8725 for <200Pa). **(d)** Distribution of ECM stiffness in WT IDH1 (*n* = 4 patient samples) and R132H IDH1 (*n* = 6 patient samples) primary LGG human patient samples as measured by AFM (two-sided Kolmogorov-Smirnov test yielded *P* = 4.8× 10^−5^). **(e)** Distribution of ECM stiffness in WT IDH1 (*n* = 6 patient samples) and R132H IDH1 (*n* = 5 patient samples) primary GBM human patient samples as measured by AFM (two-sided Kolmogorov-Smirnov test yielded *P* = 6.5×10^−5^). **(f)** Immunofluorescence images and quantification for pMLC2 (green, left) and pFAK397 (green, right) with DAPI (blue) for the tumours shown in **e** (mean ± s.e.m., *n* = 6 patient samples for WT IDH1 and *n* = 5 patient samples for R132H IDH1, two-sided unpaired *t*-test where **P = 0.007, ***P = 0.0006). Scale bars, 50 μm.

To determine whether glioma aggression associates with ECM stiffness, we utilized atomic force microscopy (AFM) to quantify the stiffness^11^ of the ECM in a cohort of fresh-frozen human brain biopsies representing non-tumour gliosis, *de novo* (primary) LGGs (WHO grades II and III) and *de novo* primary GBMs (WHO grade IV). We determined that gliotic tissue had the lowest ECM stiffness (Young's modulus, *E*;10–180 Pa) while LGGs (50–1,400 Pa) and GBMs (70–13,500 Pa) were progressively stiffer (Fig. 1a). Consistent with the elevated ECM stiffness, mechanosignalling increased progressively from gliotic tissue to the stiffer GBMs, as indicated by higher phosphorylated focal adhesion kinase (pFAK residue Tyr397) and myosin light chain 2 (pMLC2 residue Ser19) (Fig. 1b). NanoString nCounter gene expression analysis^12^ of an outcome-predictive gene cluster^13^ (aggressiveness binned on a scale of 1–10 with 1 indicating the most aggressive) of human GBM tissue biopsies indicated a significant correlation between the proportion of highly stiff areas within a GBM tissue (*E* >1,400 Pa) and worst patient prognosis score (Fig. 1c, red). By contrast, those tissues that contained a high proportion of soft ECM regions (*E* < 200 Pa) had the best patient prognosis score^13^ (Fig. 1c, blue).

Interestingly, despite a strong overall correlation between ECM stiffness and glioma grade, relatively stiff and compliant subsets of patient tumours were evident in both the LGG and GBM samples. To explore this finding, we assessed the IDH1 mutational status in our cohort of patient samples. Mutations in IDH genes characterize the majority of LGGs and a small percentage of GBMs^1–4^. For both the LGG and GBM samples, we noted that the stiffness of the ECM in the R132H IDH1 tumours was significantly less than the ECM measured in the WT IDH1 tumours (Fig. 1d,e). To this end, the R132H IDH1 GBM samples exhibited mechanical characteristics more concordant with the LGG samples (Fig.1d,e). R132H IDH1 GBM samples also had lower pFAK and pMLC2 levels, reflecting reduced mechanosignalling (Fig. 1f). These data demonstrate that ECM stiffness correlates with glioma aggression and that IDH1 mutational status associates with a softer ECM, regardless of histological grade.

### TNC modifies ECM stiffness and mechanosignalling

**Figure 2:**
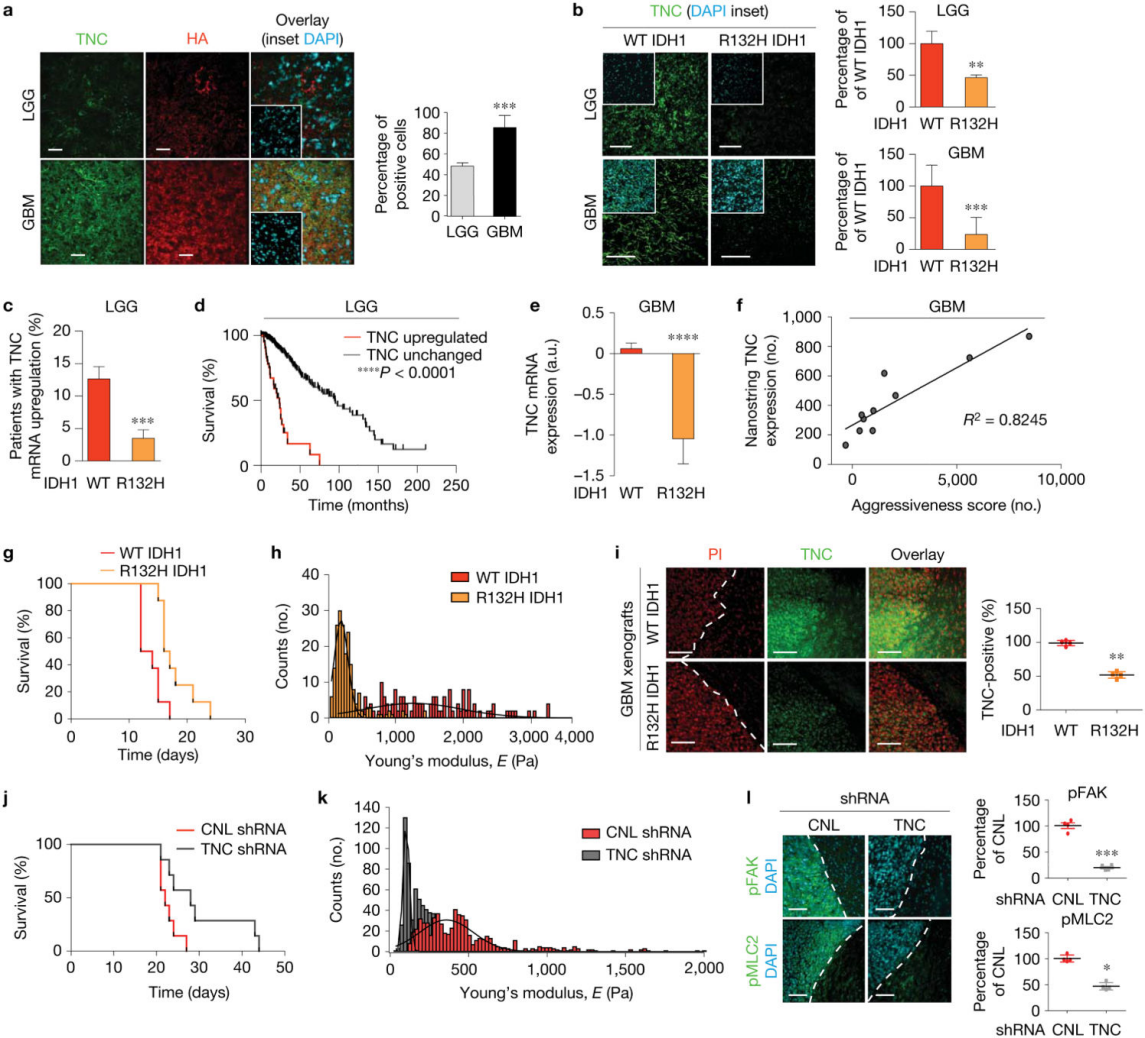
TNC modifies ECM stiffness and mechanosignalling. **(a)** Immunofluorescence images for TNC (green) and hyaluronic acid (red) with DAPI (blue), and quantification for TNC in primary LGG and primary GBM patient samples (mean ± s.e.m., *n* = 5 patient samples per group, two-sided unpaired *t*-test, ***P = 0.00032). **(b)** Immunofluorescence images and quantification for TNC with DAPI in WT IDH1 and R132H IDH1 primary LGG and primary GBM patient samples (mean ± s.e.m., *n* = 5 patient samples per group, two-sided unpaired *t*-test, **P = 0.0031 for LGG and ***P = 0.0008 for GBM). **(c)** The Cancer Genome Atlas (TCGA) data analysed for TNC upregulation in WT (*n* = 64 patients) and R132H IDH1 (*n* = 219 patients) human tumours (mean ± s.d., *z*-score threshold ±1.0, Mann–Whitney *U*-test, ***P< 0.0001). **(d)** Patient survival stratified by TNC status (TNC upregulation (*n* = 45 patients), TNC unchanged (*n* = 468 patients), *z*-score threshold ±1.0, log-rank/Mantel–Coxtest, ***P<0.0001). **(e)** TCGA data analysed for relative TNC expression in WT (*n*=199 patients) and R132H (*n*=12 patients) IDH1 human tumours (mean ± s.d., Wilcox/Mann–Whitney *U*-test, ***P=4× 10^−4^). **(f)** NanoString nCounter gene expression analysis of tumours from Fig. 1c correlating TNC expression with raw NanoString nCounter aggressiveness scores (higher score indicating higher aggressiveness, *n* = 10 patient samples, linear regression where *R*^2^ = 0.8245). **(g)** Survival of xenograft mice injected with either WT IDH1 (red, *n* = 8 mice) or R132H IDH1 (orange, *n* = 8 mice) human GBM cells (log-rank (Mantel–Cox) test, *P* = 0.04). **(h)** Distribution of ECM stiffness in xenograft tumours from human GBM cells infected with either WT or R132H IDH1 cells (*n* = 4 mice per group, *n* was used to derive statistics, histogram encompasses all measurements across the five regions of four mice, two-sided Kolmogorov-Smirnov test, *P*=1.3×10^−3^). **(i)** Immunofluorescence images and quantification for TNC with propidium iodide (PI) in xenograft tumours derived from either WT or R132H IDH1 human GBM cells (mean ± s.e.m., *n* = 4 WT mice, *n* = 5 R132H IDH1 mice, two-sided unpaired *t*-test, **P = 0.00023). Dashed lines indicate tumour edges. **(j)** Survival of xenograft mice injected with WT IDH1 human GBM cells expressing either a control (CNL) scramble shRNA (red) or an shRNA targeting TNC (grey) (*n* = 8 mice per group, log-rank (Mantel–Cox) test, *P* = 0.03). **(k)** Distribution of ECM stiffness in xenograft tumours derived from WT IDH1 primary human GBM cells expressing either a control scramble shRNA (red) or shRNA targeting TNC (grey) (*n* = 6 mice per group, *n* was used to derive statistics, histogram encompasses all the measurements across all regions of all six mice, two-sided Kolmogorov-Smirnov test, *P*= 1.73 × 10^−2^). **(l)** Immunofluorescence images and quantification for pFAK397 (***P = 0.0008) and pMLC2 (*P = 0.009) with DAPI in xenograft tumours expressing control scramble shRNA or shRNA targeting TNC (mean ± s.e.m., *n* = 4 mice per group, two-sided unpaired *t*-test). Scale bar, 50 μm.

To examine the relationship between IDH mutation and ECM stiffness, we used a candidate approach to profile major ECM constituents in GBM, hyaluronic acid (HA), and HA-interacting molecules^14,15^. Immunohistochemistry revealed that the elevated ECM stiffness in the patients with poorer prognosis was accompanied by a substantial increase in HA expression as well as increased levels of TNC, and indicated that these levels increased progressively from gliosis to LGGs and to the GBMs (Fig. 2a and Supplementary Fig. 2a,b)^16^. Importantly, ECM stiffness did not correlate with levels or distribution of type I collagen, vasculature or cellularity (Supplementary Fig. 1a–g). Indeed, a survey of the ECM status of the softer ECMs in the IDH1-mutant human GBM biopsies revealed a reduction in the levels of TNC (Fig. 2b). Further, bioinformatics analysis of publicly available messenger RNA expression data^1^ revealed a significant correlation between IDH1 mutational status and TNC expression in LGG tumours, with stratification of LGG patients by TNC upregulation revealing reduced patient survival (Fig. 2c,d).

Similarly, IDH1 mutational status correlated significantly with TNC mRNA expression in GBM^17,18^ tumours (Fig. 2e). NanoString nCounter gene expression analysis^12^ of TNC expression (where lower scores correspond to lower expression) in GBM samples yielded a significant correlation between TNC and the tumour aggressiveness score derived via the outcome-predictive gene expression signature (where lower scores correlated with reduced overall survival, Fig. 2f).

Consistent with a relationship between IDH1 mutational status, ECM stiffness and glioma aggression, ectopic expression of the R132H IDH1 mutation significantly improved the survival of nude mice bearing xenografts derived from either primary GBM cells or the U87 cell line (Fig. 2g and Supplementary Fig. 2c,d). The resultant tumours were also softer and exhibited reduced mechanosignalling, as revealed by decreased pMLC2 and pFAK (Fig. 2h and Supplementary Fig. 2e,f). In both the primary and U87 xenograft models, we noted that the softer IDH1-mutant-expressing GBM tumours had low to negligible TNC expression (Fig. 2i and Supplementary Fig. 2g).

To experimentally test the role of TNC in ECM stiffness and glioma aggression, we knocked down TNC in primary GBM cells (WT IDH1, Supplementary Fig. 2h,i) and assessed the resulting effects on ECM stiffness and tumour aggression. Nude mice injected orthotopically with GBM cells with short hairpin RNA (shRNA)-reduced TNC not only survived longer (Fig. 2j), but the tumours were softer and had lower mechanosignalling, as revealed by reduced pFAK and pMLC2 (Fig. 2k,l). These findings demonstrate that TNC can impact glioma aggression by increasing ECM stiffness and suggest a molecular role for IDH1 mutational status in perturbing this relationship.

### HIF1α directly regulates TNC expression

**Figure 3:**
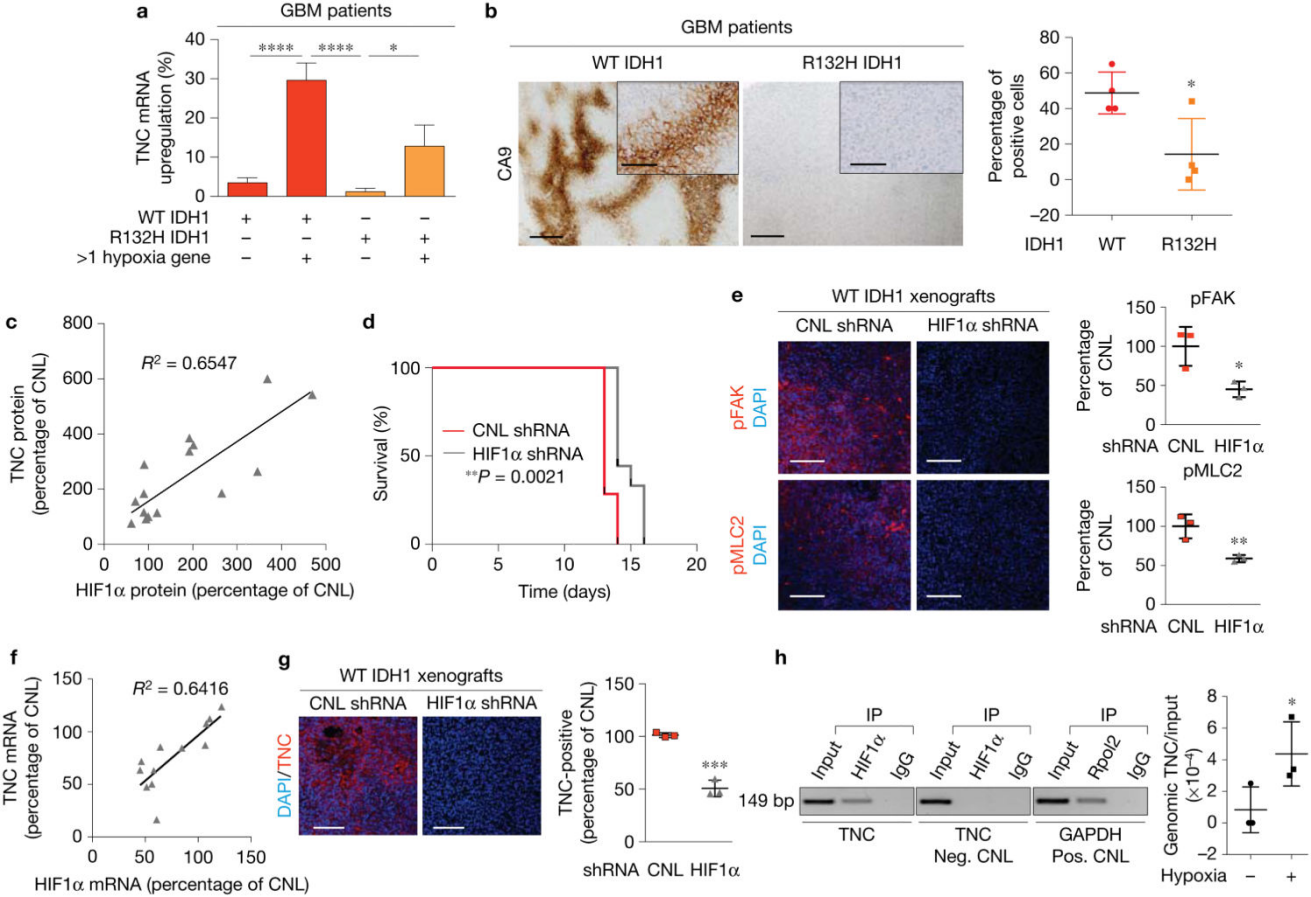
HIF1α directly regulates TNC expression. **(a)** Analysis of publicly available TCGA data analysed for TNC upregulation in WT IDH1 patient tumours expressing >1 hypoxia gene (n=108 patients) or ≤1 hypoxia gene (*n* = 201 patients), and R132H IDH1 patient tumours expressing >1 hypoxia gene (*n* = 39 patients) or ≤1 hypoxia gene (n=160 patients) (mean ± s.d., *z*-score threshold ±1.0, one-way ANOVA with Dunn's multiple comparisons test where ***P< 0.0001, *P<0.05). **(b)** Immunohistochemical analysis and quantification of percentage of CA9-positive tumour cells in IDH1 WT (red) versus IDH1 R132H (orange) GBM patient biopsies (mean ± s.e.m., *n* = 4 patient tissues per group, two-sided unpaired *t*-test, *P = 0.0437). Inset scale bar, 15μm. **(c)** Correlation between TNC protein and HIF1α protein in WT IDH1 human GBM cells cultured *in vitro* (*n*=17 biological replicate samples, linear regression where *R*^2^ = 0.6547 and *P*< 0.0001). **(d)** Kaplan-Meier graph showing survival of xenograft mice injected with primary human GBM cells expressing either a control scramble shRNA (red, *n* = 7 mice) or an shRNA targeting HIF1α (grey, *n* = 9 mice) (log-rank (Mantel–Cox) test, **P = 0.0021). **(e)** Immunofluorescence images and quantification for pFAK397 (red, top, *P< 0.0239) and pMLC2 (red, bottom, **P<0.0114) with DAPI (blue) in xenograft WT IDH1 tumours expressing control scramble shRNA or shRNAtargeting HIF1α (mean ± s.d., *n*=3 mice per group). **(f)** Correlation graph between TNC mRNA expression and HIF1α mRNA in WT IDH1 human GBM cells (n=12 biological replicate samples, linear regression where *R*^2^ = 0.6416, *P*< 0.0001). **(g)** Immunofluorescence images and quantification for TNC (red) in xenograft tumours derived from WT IDH1 primary human GBM cells expressing either a control scramble shRNA or an shRNA targeting HIF1α (mean ± s.d., *n* = 3 mice per group, two-sided unpaired *t*-test, ***P = 0.0004). **(h)** Representative gel of chromatin immunoprecipitation studies in R132H IDH1 primary human cells demonstratingthe immunoprecipitation (IP) of HIF1α with the TNC promoter (Neg. CNL denotes negative control, Pos. CNL denotes positive control and Rpol2 denotes RNA polymerase II; mean ± s.e.m., *n*=3 biological replicate samples including three intronic primer sets, two-sided unpaired *t*-test, *P = 0.0284). Scale bars, 50μm unless otherwise indicated. Unprocessed original scans of blots are shown in Supplementary Fig. 7.

Interestingly, we noted that compliant ECM regions in xenograft tumours with TNC knocked down (Fig. 2j–l) had reduced HIF1α expression^19^ (Supplementary Fig. 3a,b). Previous work implicated hypoxia and HIF1α signalling in TNC expression^20,21^. A bioinformatics analysis revealed a strong correlation between TNC mRNA upregulation and expression of hypoxia-induced genes in both WT and R132H IDH1 patient GBMs (Fig. 3a), and expression of carbonic anhydrase 9 (CA9), a HIF1α-target gene, was reduced in R132H tumours (Fig. 3b). Accordingly, we explored the relationship between GBM aggression and HIF1α-induced TNC expression.

TNC protein expression increased in (WT IDH1) human GBM cells cultured *in vitro* under hypoxia (Fig. 3c and Supplementary Fig. 3d,e). shRNA-mediated knockdown of HIF1α reduced hypoxia-dependent induction of TNC (SupplementaryFig.3c–f). To investigate the role of reduced HIF1α expression *in vivo*, we orthotopically injected the WT IDH1 primary GBM cells with shRNA-reduced HIF1α. Consistent with a direct link between HIF1α and TNC expression, reducing HIF1α expression increased survival and reduced mechanosignalling (Fig. 3d,e). Importantly, reducing HIF1α expression significantly correlated with reduced TNC mRNA and protein expression levels (Fig. 3f,g).

To determine whether HIF1α directly regulates TNC expression, we identified putative hypoxia-response elements in the promoter of TNC (provided in the Methods). We performed chromatin immunoprecipitation assays using HIF1α as the bait in WT IDH1 human GBM cells and found that HIF1α binds directly to the TNC promoter as indicated by co-precipitation of HIF1α with the TNC promoter (Fig. 3h). As expected, this interaction was largely dependent on HIF1α stabilization under reduced oxygen tension (1% O_2_, hypoxia, Fig. 3h). These data suggest that tissue mechanics and HIF1α could modify glioma aggression via a positive feedback loop.

### R132H IDH1 primary GBMs cannot tune HIF1α

**Figure 4:**
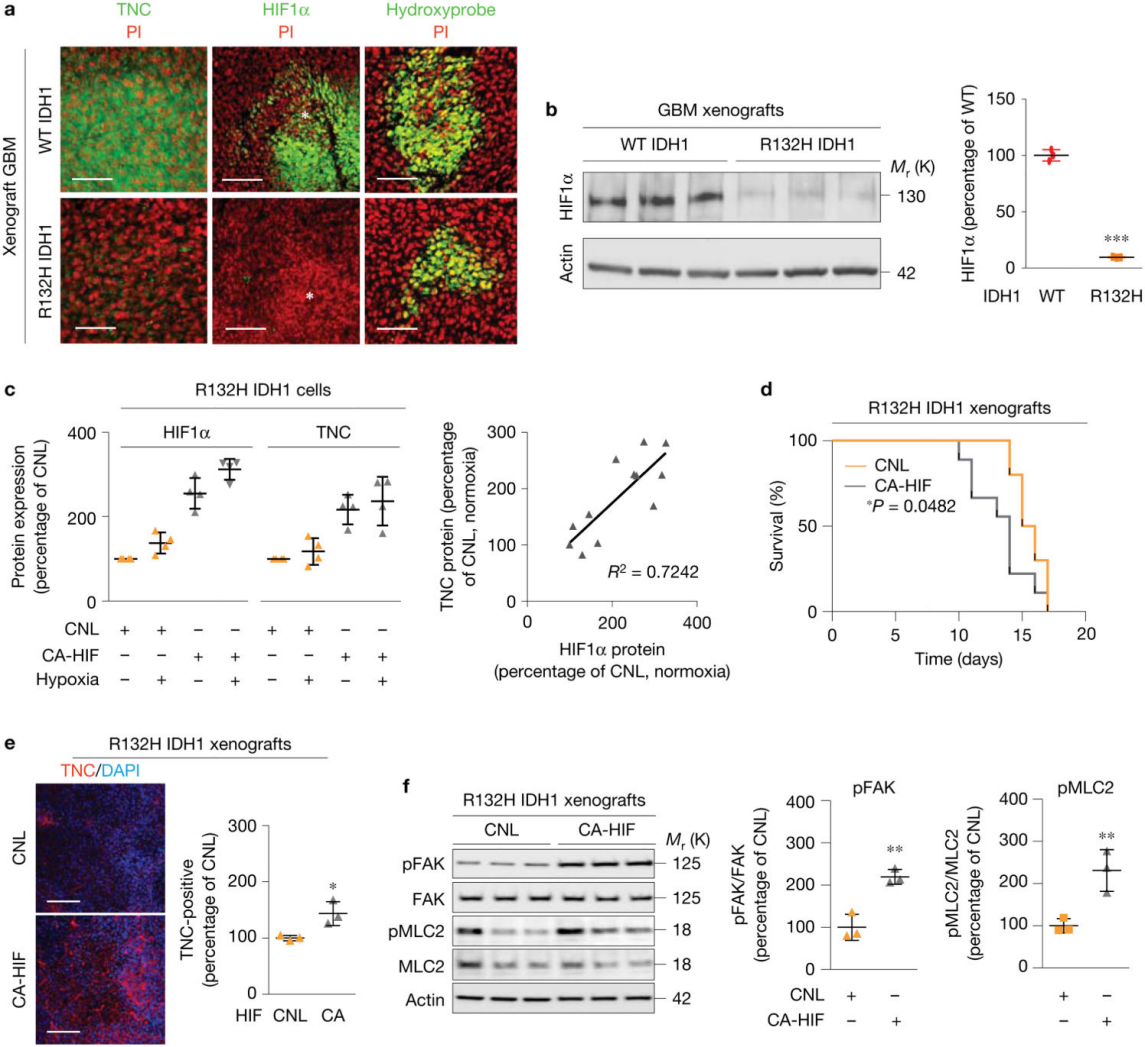
R132H IDH1 primary GBMs cannot tune HIF1α. **(a)** Immunofluorescence images of xenograft tumours derived from primary human GBM cells expressing either WT or R132H IDH1 immunostained for TNC (green, left), HIF1α (green, middle), and hypoxyprobe (green, right) with PI (red); asterisks indicate areas of necrosis. **(b)** Immunoblot and quantification of xenograft tumours derived from either WT or R132H IDH1 primary human GBM cells. Results were normalized to β-actin and graphed relative to WT IDH1 expression (mean ± s.d., *n* = 3 mice per group, two-sided unpaired *t*-test, ***P< 0.001). **(c)** Left: quantification of HIF1α (left) and TNC (right) protein expression under hypoxia and normoxia in R132H IDH1 cells expressing either a control scramble (CNL, orange) or a constitutively active HIF1α (CA-HIF, grey) construct (mean ± s.e.m., *n*=4 biological replicate samples per group, one-way ANOVA with Tukey's multiple comparisons test, *P*< 0.0001). Right: correlation between TNC protein expression and HIF1α protein in R132H IDH1 tumours expressing the constitutively active HIF1α (CA-HIF) (*n* = 15 biological replicates, linear regression where *R*^2^ = 0.7242, *P*< 0.0001). **(d)** Kaplan-Meier graph showing survival of xenograft mice injected with R132H IDH1 primary human GBM cells expressing either a vector control (CNL, orange, *n*=10 mice) or a constitutively active HIF1α (CA-HIF, grey, *n* = 9 mice, log-rank (Mantel–Cox) test, *P = 0.0482). **(e)** Immunofluorescence images and quantification for TNC (red) in xenograft tumours derived from either CNL or CA-HIF-expressing R132H IDH1 primary human GBM cells (mean ± s.d., *n* = 3 mice per group, two-sided unpaired *t*-test, *P = 0.0260). **(f)** Immunoblot and quantification of pFAK397 (*n* = 3 mice per group, two-sided unpaired *t*-test, **P < 0.0044) and pMLC2 (mean ± s.d., *n* = 3 mice per group, two-sided unpaired *t*-test, **P < 0.0121) protein expression in CNL and CA-HIF1α xenografts normalized to total FAK and MLC2, respectively, and β-actin loading control, and represented as percentage of expression of CNL tumours (n=3 mice per group). Scale bars, 50μm. Unprocessed original scans of blots are shown in Supplementary Fig. 7.

The presence of the IDH1 mutation could enhance GBM patient survival by increasing the production of the onco-metabolite (*R*)-2-hydroxyglutarate, which can induce genome-wide methylation alterations and alter cellular redox state to promote cellular transformation^5,22^. IDH1 could also influence tumour phenotype by regulating HIF1α (refs 6,7,23,24). Analysis of WT and R132H IDH1 GBM xenografts revealed that R132H IDH1 tumours had low to barely detectable nuclear HIF1α (and TNC) despite evident necrosis (asterisks) and hypoxia (Fig. 4a and Supplementary Fig. 3g).

Immunoblot analysis confirmed that the IDH1-mutant GBMs had significantly reduced total tissue HIF1α protein (Fig. 4b) suggesting a profound dysregulation of the hypoxic response in xenograft tumours expressing the R132H IDH1 mutation.

Experimentally, we observed a lack of upregulation of either HIF1α or TNC in R132H IDH1 GBM cells cultured *in vitro* under hypoxia, a finding that was recapitulated with experimental models endogenously expressing R132H IDH1 (Fig. 4c and Supplementary Fig. 4a–c). When we ectopically expressed a constitutively active HIF1α (CA-HIF)^25^ in the R132H IDH1 cells, we found a robust upregulation of TNC expression both under normoxia and hypoxia (Fig. 4c and Supplementary Fig. 4c). Importantly, survival was decreased in mice bearing orthotopic R132H IDH1 GBM tumours expressing the constitutively active HIF1α (CA-HIF) compared with vector-only tumours (Fig. 4d). Consistently, both mechanosignalling and TNC were elevated in constitutively active HIF1α tumours (Fig. 4e, f). These data suggest that the R132H IDH1 mutation blunts hypoxia sensing to reduce HIF1α-dependent TNC expression, and provide a potential explanation for why patients bearing tumours with mutations in IDH experience improved survival.

### Mechanosignalling promotes R132H IDH1 tumour aggression

**Figure 5:**
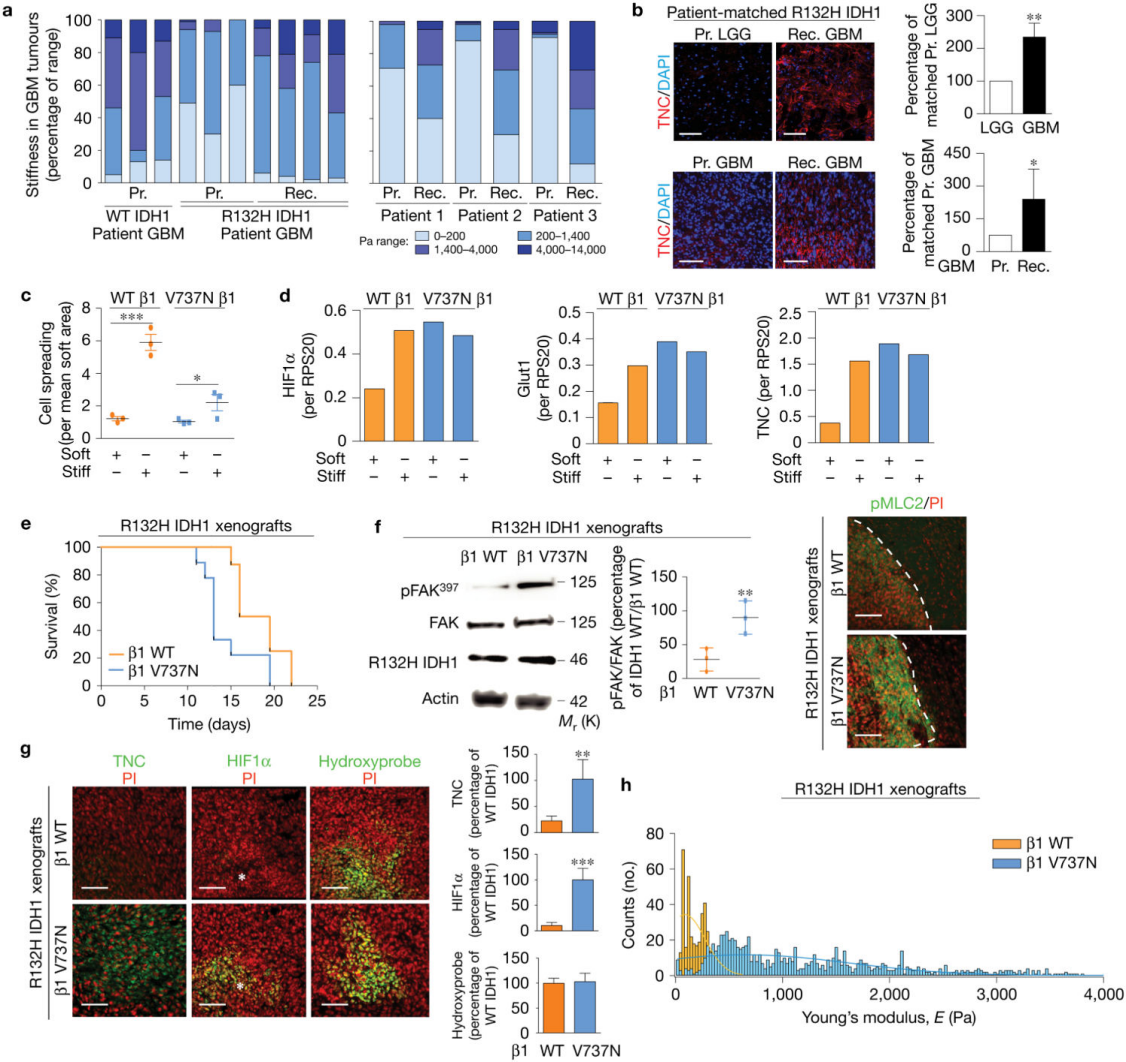
Mechanosignalling promotes R132H IDH1 tumour aggression. **(a)** Left: ECM stiffness in primary (Pr.) WT IDH1 (*n* = 3), primary R132H IDH1 (*n* = 3), and recurrent (Rec.) secondary R132H IDH1 (treated with temozolomide and radiation, *n* = 4) GBM human tumours (two-sided Kolmogorov-Smirnov test between primary and recurrent R132H IDH1 tumours, *P* = 0.21 × 10^−1^). Right: ECM stiffness in matched pairs of R132H IDH1 primary and pos*t*-treatment secondary GBM human tumours (*n* = 3 patients per group, two-sided Kolmogorov-Smirnov, *P* = 2.11 × 10^−3^). **(b)** Immunofluorescence images and quantification for TNC in primary and recurrent secondary R132H IDH1 tumours (mean ± s.e.m., *n* = 5 patients per group, two-sided unpaired Mann-Whitney test, **P = 0.0079 (top) and *P = 0.0392 (bottom)). **(c)** Cell spreading area for R132H IDH1/WT β1 (orange) and R132H IDH1/V737N β1 (blue) primary human GBM cells grown on soft (140 Pa) or stiff (6,000 Pa) polyacrylamide (PA) gels (mean ± s.e.m., *n* = 3 biological replicate samples per group, one-way ANOVA with Tukey's multiple comparisons, ***P= 1.09 × 10^−2^, *P<0.05). **(d)** HIF1α mRNA expression (left), Glut1 (middle), and TNC (right) for R132H IDH1/WT β1 (orange) and R132H IDH1/V737N β1 (blue) primary human GBM cells cultured on soft or stiff PA gels. Expression was normalized to RPS20 ribosomal RNA, which itself was not changed by ECM stiffness (graph shows the average of two biological replicate samples). **(e)** Survival of mice xenografted with R132H IDH1 primary human GBM cells expressing either WT β1 or V737N β1 (*n* = 8 mice per group, log-rank (Mantel–Cox) test, *P*<0.02). **(f)** Left: immunoblot and quantification of FAK activity (pFAK^397^) in primary R132H IDH1 primary human GBM cells expressing either WT β1 (orange) or V737N β1 (blue). Results were normalized to total FAK (mean ± s.d., *n* = 3 mice per group, two-sided unpaired *t*-test, **P = 0.0229). Right: immunofluorescence imagesofxenografttumours derived from primary human R132H IDH1 primary human GBM cells expressing either WT β1 or V737N β1, and immunostained for pMLCwith PI. Dashed lines indicate tumour edges. **(g)** Immunofluorescence images of xenograft tumours derived from R132H IDH1 primary human GBM cells expressing either WT β1 or V737N β1 immunostained for TNC (left), HIF1α (middle), and hypoxyprobe (right) with PI; asterisks indicate areas of necrosis. Immunofluorescence was quantified as the percentage of WT IDH1/WT β1 (mean ± s.d., *n* = 6 mice per group, two-sided unpaired *t*-test, **P = 0.009 and ***P = 0.0002). **(h)** ECM stiffness in xenograft tumours derived from either WT β1 or V737N β1 R132H IDH1 primary human GBM cells (*n* = 8 mice per group, two-sided Kolmogorov-Smirnov test, *P* = 4.87 × 10^−5^). Scale bars, 50 [Am. Unprocessed original scans of blots are shown in Supplementary Fig. 7.

While IDH mutations may confer prolonged survival in glioma patients suffering from gliomas, IDH-mutation-bearing gliomas still frequently recur despite surgical resection and treatment^26,27^. Standard of care for GBM patients involves surgical resection followed by high-dose radiation treatment and chemotherapy. These treatment modalities can cause dramatic changes in the post-treatment tumour microenvironment, including the deposition and remodelling of ECM molecules, which we and others have shown to be associated with stromal stiffening^28^. Accordingly, we asked whether the post-treatment (secondary) IDH1-mutant GBMs were stiffer than the patient-matched primary LGG or GBM tumours. AFM analysis of secondary IDH1-mutant patient GBMs revealed highly stiff ECMs that were significantly stiffer than those measured in primary IDH1-mutant GBMs and were as stiff, if not stiffer, than those measured in primary WT IDH1 GBMs (Fig. 5a left compared with Fig. 1e). Indeed, mechanical analysis of patient-matched IDH1-mutant GBM pairs, at initial diagnosis (primary tumour) and at recurrence (secondary tumour excised after treatment with gamma radiation and temozolomide chemotherapy), revealed a remarkable increase in the stiffness of the associated ECMs in the secondary IDH1-mutant GBMs (Fig. 5a, right). Further, we noted a strong increase in TNC expression in secondary tumours for both LGG and GBM patient cohorts (Fig. 5b).

As such, we explored whether we could increase the aggression of R132H IDH1 GBM cells by enhancing mechanosignalling through plating the cells on a stiff substrate. We plated the R132H IDH1 GBM cells on two-dimensional polyacrylamide (PA) gels with a calibrated stiffness ranging from very soft (140 Pa, representing normal brain^29,30^) to stiff (6,000 Pa, representing the upper range of elastic moduli in GBM samples, as presented in Fig. 1d). We compared their characteristic cell spreading behaviour and found that R132H IDH1 cells spread better on stiff substrates (Fig. 5c). When R132H IDH1 cells were subjected to hypoxia, they showed a robust upregulation of HIF1A, TNC and Glut1 expression (Fig. 5d). By contrast, when plated on a soft substrate analogous to the soft ECMs we measured *in vivo*, they showed a blunted hypoxia sensing response (Fig. 5d). These findings indicate that a stiffened ECM overrides the blunted hypoxia sensing conferred by expression of the mutant R132H IDH1.

To directly assess whether enhancing mechanosignalling could override the blunted hypoxia sensing conferred by the R132H IDH1 gene, we ectopically expressed an auto-clustering β1 integrin mutant (V737N) in the R132H IDH1 cells to recapitulate downstream stiffness-dependent mechanosignalling through elevation of FAK signalling^29^. We found that R132H IDH1 GBM cells expressing the auto-clustering V737N mutated β1 integrin spread similarly on the soft and stiff substrates (Fig. 5c). Consistent with cell spreading, V737N R132H IDH1 GBM cells responded to hypoxia largely independently of the underlying substrate stiffness with robust increases in HIF1α, TNC and Glut1 expression levels on both soft and stiff substrates (Fig. 5d). These data indicate that ECM stiffness can induce HIF1α and suggest that the relationship between IDH1 mutation and hypoxia sensing through HIF1α may be largely dependent on the stiffness of the ECM micro environment of the tissue.

We performed xenograft injections of R132H IDH1 cells expressing either WT or V737N β1 integrin to test whether enhancing cellular mechanosignalling in mutant GBMs would increase tumour aggression and, if so, whether this increase would be associated with elevated HIF1α and TNC expression. Survival was decreased in mice bearing the V737N β1 tumours compared with WT β1 tumours (Fig. 5e). Tumours derived from R132H IDH1 GBM cells expressing the V737N integrin demonstrated increased mechanosignalling (Fig. 5f), and expressed elevated levels of both HIF1α and TNC (Fig. 5g). In contrast, R132H IDH1 GBM tumours expressing WT β1 integrin had low to negligible HIF1α and TNC protein expression, despite pronounced hypoxia (Fig. 5g). Interestingly, V737N tumours were substantially stiffer than control tumours (Fig. 5h), suggesting a positive feedback whereby elevated mechanosignalling increases TNC expression to increase ECM stiffness. These findings further link GBM aggression to tissue mechanics, and suggest that elevated mechanosignalling can bypass the protective activity imparted by the R132H IDH1 to promote tumour aggression.

### miR-203 targets HIF1α and TNC

**Figure 6:**
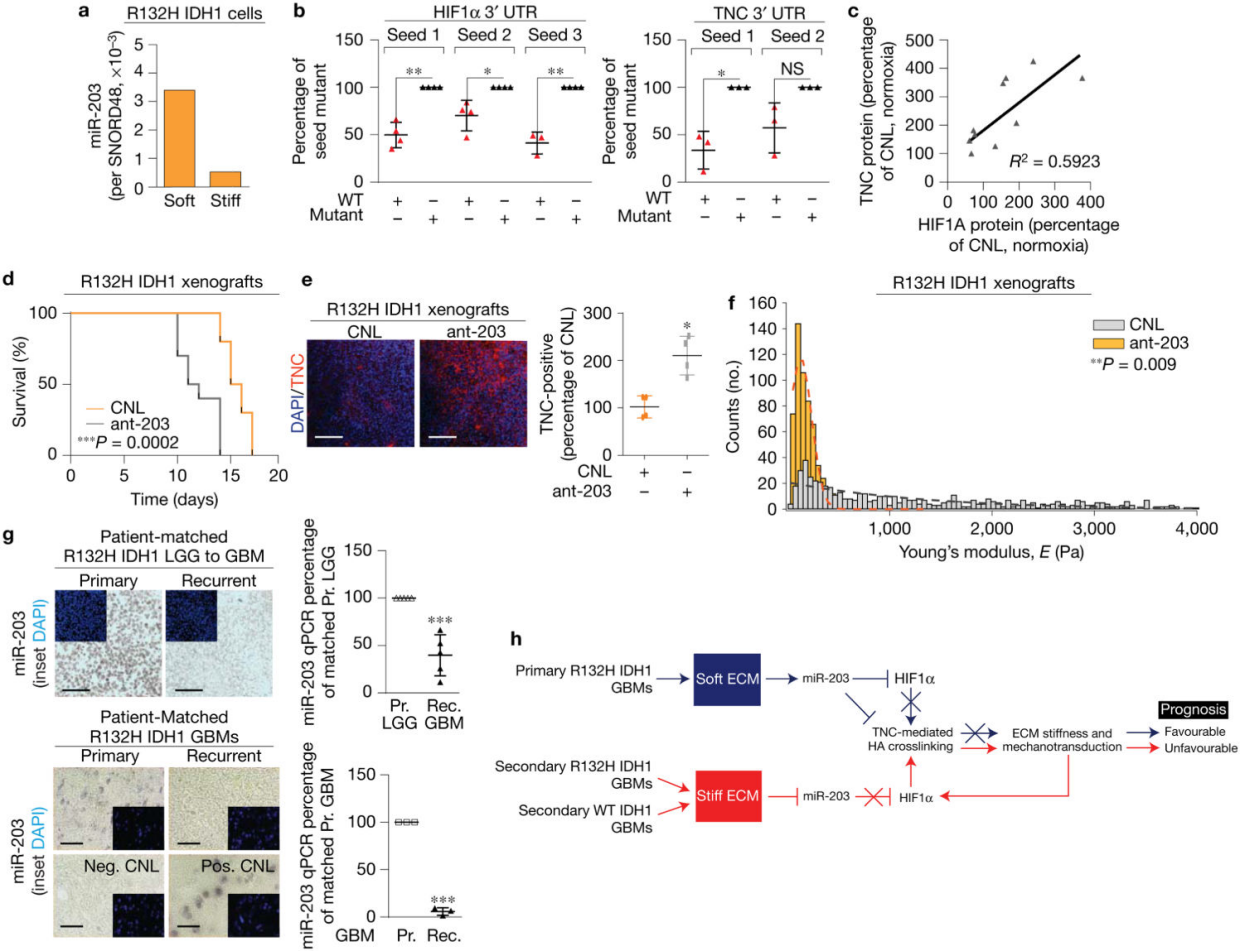
miR-203 targets HIF1α and TNC. (a) miR-203 expression in R132H IDH1 primary human GBM cells cultured on soft or stiff PA gels *in vitro* normalized to SNORD48 small nucleolar RNA (graph shows the mean of two biological replicate samples). (b) Luciferase reporter assay performed on WT IDH1 cells using the wild-type (red) and mutated (black) 3′UTR consensus sites on HIF1α mRNA (left, three seed sequences) and on TNC mRNA (right, two seed sequences) normalized by secreted alkaline phosphatase levels; each group is graphed relative to its respective mutated group (mean ± s.e.m., *n* = 4 biological replicate samples, two-sided paired *t*-test, for HIF1α: **P = 0.0048, seed 1; *P = 0.0343, seed 2; **P = 0.0128, seed 3; for TNC: *P = 0.0286, seed 1; NS (not significant) *P* = 0.1071, seed 2). (c) Correlation between TNC and HIF1α protein expression levels in R132H IDH1 human GBM cells with reduced miR-203 (ant-203) compared with vector controls (CNL) cultured *in vitro* under hypoxia (n=12 biological replicate samples, linear regression *R*^2^ = 0.5923, P<0.0034). (d) Kaplan-Meier graph showing survival of mice xenografted with R132H IDH1 GBM cells expressing CNL (orange, n=10 mice) or ant-203 (grey, n=10 mice) (log-rank (Mantel–Cox) test, ***P = 0.0002). Note, the same vector control cohort is shown in Fig. 4d-f. (e) Immunofluorescence images and quantification for TNC (red) in xenograft tumours derived from primary human GBM cells expressing either CNL or ant-203 vectors (mean ± s.d., *n* = 4 mice per group, two-sided unpaired *t*-test, *P = 0.0491). (f) Histogram showing the distribution of ECM stiffness in xenograft tumours derived from primary R132H IDH1 human GBM cells expressing either a CNL or ant-203 constructs (n = 5 mice per group, *n* was used to derive statistics, two-sided Kolmogorov-Smirnov test, **P<0.009). (g) Bright-field images (left) of miR-203 *in situ* hybridization in patient-matched primary and recurrent secondary R132H IDH1 patient glioma tumours. Top: patient-matched primary LGG and secondary GBM. Bottom: patient-matched primary GBM and secondary GBM, with corresponding U6-positive control and scramble negative control. Insets show staining with DAPI. Right: qPCR quantification of miR-203 expression in the patient samples shown on the left; miR-203 expression was normalized by SNORD48 small nucleolar RNA expression (mean ± s.d., two-sided paired *t*-test, for LGG to GBM, *n* = 5 patients and ***P = 0.0034; for GBM to GBM, *n* = 3 patients and ***P = 0.0006). (h) Graphic depicting a model of ECM stiffness-driven repression of miR-203 expression to activate HIF1α/TNC. Scale bars, 50 μm.

Next, we explored the putative positive feedback whereby elevated mechanosignalling increases TNC expression to increase ECM stiffness. We previously found that ECM stiffness regulates microRNA expression to alter tumour progression, including miR-203, a microRNA implicated in GBM aggression, recurrence, and treatment responsiveness^9,31–34^. We found that R132H IDH1 cells expressed fivefold higher levels of miR-203 on soft PA gels compared with stiff gels (Fig. 6a). Further, we identified several putative consensus sites in the 3′ UTRs of both HIF1α and TNC mRNA (Supplementary Fig. 5a). Consistent with the hypothesis that tissue mechanics may induce HIF1α and TNC in GBMs expressing the mutant IDH1 by reducing levels of miR-203, reporter assays using the wild-type and mutated 3′UTR consensus sites confirmed that miR-203 interacts with and inhibits HIF1α transcription at three out of three predicted sites and TNC at one of two predicted sites (Fig. 6b). Experimentally, we tested whether antagomiR-mediated knockdown^35^ of miR-203 could elevate HIF1α and TNC levels in R132H IDH1 GBM cells grown in culture (on a soft ECM where miR-203 levels are elevated) and *in vivo* (Supplementary Fig. 5b). Consistent with a mechanistic interaction, we observed elevated HIF1α and TNC levels in R132H IDH1 GBM cells with reduced miR-203 (ant-203) compared with vector controls (Supplementary Fig. 5c,d), with a significant correlation between their protein expression (Fig. 6c). Importantly, orthotopically injected R132H IDH1 tumours with reduced miR-203 exhibited decreased survival (Fig. 6d), increased TNC expression (Fig. 6e), and elevated ECM stiffness (Fig. 6f).

Next, we assessed the expression of miR-203 in our patient-matched primary and secondary tumours, using *in situ* and quantitative PCR, and found that the stiffer secondary tumours exhibited reduced miR-203 expression compared with their patient-matched primary tumour counterparts for patients who originally presented with either LGG or GBM tumours (Figs 5a and 6g). Taken together, these data suggest that tumour therapy may contribute to the development of more aggressive IDH1-mutant GBMs by increasing ECM stiffness and reducing miR-203 expression (Fig. 6h).

## Discussion

Here, we establish a clinically relevant role for ECM mechanics in glioma aggression. We link HIF1α-dependent hypoxia sensing and TNC expression with an aggressive tumour phenotype, and demonstrate that ECM stiffness directly represses miR-203 expression to activate HIF1α-dependent TNC deposition via a positive feedback loop. The relationship between IDH1 mutations and HIF1α is controversial^6,7,24,36,37^. Our studies highlight the cellular plasticity and sensitivity of R132H IDH1 GBM cells to ECM stiffness and oxygen tension. In all likelihood, mutations in IDH1 will exert a myriad of context-dependent effects to varying consequence depending on the origin of the tumour, the stage of the disease, the oxygen tension within the tumour and the duration of the disease. Gain-of-function IDH mutations often induce a CpG island methylator phenotype (G-CIMP)^22,38^ that is associated with improved outcome^38^. IDH mutations are most prevalent in LGG, which, as a result of being less proliferative and diffusely infiltrative, may be less hypoxic than higher-grade GBMs^1,39,40^. Therefore, LGGs frequently have a relatively long disease course where the long-term effect of IDH mutations (global DNA hypermethylation) is likely to dominate LGG pathobiology. On the contrary, for the rapidly progressing GBMs, the acute effects of IDH1 mutation may profoundly dictate GBM cell behaviour. Our work presents an example of one mechanism, outside of the sustained effects imparted by hypermethylation, whereby expression of the mutant IDH1 can compromise HIF1α induction in response to hypoxia to modify the ECM and alter tissue mechanics and tumour aggression. Our findings suggest that ECM mechanics provides putative promising targets for therapeutic interventions aimed at disabling biomechanically driven pathways that intersect with glioma aggression to enhance treatment efficacy, delay disease progression, and improve patient survival.

## Methods

### Human samples

Human tissue samples, lacking any patient-identifying information, were either collected under approved study (protocol number 11-07588) and in accordance with the University of California, San Francisco Committee on Human Research policy, or obtained from the University of California, San Francisco Brain Tissue Bank. All human tissue samples were collected in compliance with informed consent policy. Being fully aware of the histopathological heterogeneity of glioblastoma ‘multiforme’, we performed a meticulous region-to-region analysis correlating the mechanical properties of the patient gliosis and tumour samples underlying Fig. 1. We worked with a pathologist to identify specific regions within the patient samples to correlate ECM stiffness with mechanosignalling in adjacent sections. In H&E-stained serial sections, distinct regions (varying from approximately 1–3 mm^2^ in area) of the patient tumours were pathologist identified, with care taken to avoid vasculature and adjacent non-malignant tissue. Stiffness (as measured by AFM), immunostaining and *in situ* hybridization analyses were performed in region-matched adjacent serial sections.

### Mouse studies

All mice were maintained in accordance with University of California Institutional Animal Care and Use Committee (IACUC) guidelines under protocol number AN109372-01. For all mouse xenograft studies, an hour before intracranial injection, cells were washed once with PBS solution, collected by trypsinization, counted, and re-suspended in PBS at 100,000 cells per microlitre. For intracranial injections, 5–6-week-old NCR nude female mice were anaesthetized with 2% isoflurane, injected subcutaneously with buprenorphine (0.03 mgkg^−1^), and slowly injected with a 3 μl tumour cell suspension (300,000 cells per 3 μl injection) into the striatum, as described previously^41^. For TNC shRNA studies, TNC knockdown was induced by addition of 20 mM IPTG in the drinking water of mice for the duration of the entire experiment. Mice were euthanized when exhibiting 15% weight loss. For hypoxia experiments, mice were injected via intraperitoneal injection with 60 mg kg^−1^ hypoxyprobe 70 min prior to euthanization.

### Immunostaining and immunoblotting

Immunofluorescence and immuno blotting analysis were performed as described previously^29,30^. For all immunoblotting, cells were lysed in RIPA or Laemmli buffer^42^. Antibodies used for immunofluorescence and immunoblotting analysis are listed below.

### Antibodies and reagents

Antibodies were as follows (used at 1μg ml^−1^ for western blotting and chromatin immunoprecipitation (ChIP) and at indicated concentrations for immunofluorescence studies): tenascin C (Abcam ab108930; 1:1,000), pY397-FAK (Invitrogen 44625; 1:200), total FAK (BD Biosciences 610088), pMLC2 (Cell Signaling 3671; 1:200), total MLC2 (Abcam ab92721, clone EPR3741; 1:200), p-MyPT1 (Millipore ABS45; 1:200), hyaluronic acid binding protein (Calbiochem, 385911; 1:500), aggrecan (Abcam ab3778; 1:500), versican (Abcam ab19345; 1:500), collagen 1 (Abcam ab34710; 1:1,000), propidium iodide (AcrosOrganics 440300250; 1 μg ml^−1^), β-actin (Sigma-Aldrich a5441), HIF1α (Abcam ab-1, for immunofluorescence; 1:200; Abcam ab1, for ChIP; Novus 100-449, for western blotting), hypoxyprobe (Hypoxyprobe, HP1-100 Kit; per manual), CD31 (BD 550389; 1:500), laminin (Abcam ab11575; 1:500), RNA polymerase II (Millipore 05-623B), rabbit IgG isotype control (Cell Signaling 2729; 1:500), Alexa Fluor-conjugated goat secondary anti-mouse IgG and anti-rabbit IgG antibodies (Invitrogen A11012 and A11005; 1:500) and HRP-conjugated rabbit secondary antibody (GE Healthcare Life Sciences NA934VS; 1:5,000).

### Cell culture conditions

All cells were maintained at 37 °C and 5% CO_2_. Primary human GBM cells (GBM43) have been previously described^43^, and were cultured in Neurobasal-A media (Invitrogen) supplemented with B27 Supplement (Invitrogen), N2 Supplement (Invitrogen), 20ngml^_1^ epidermal growth factor (Peprotech), 20ngml^−1^ fibroblast growth factor (Peprotech), and 100 units ml^−1^ penicillin/streptomycin. U87 cells (obtained from and authenticated by ATCC) were grown in DMEM supplemented with 10% fetal bovine serum (Hyclone), 2mM L-glutamine, and 100 units ml^−1^ penicillin/streptomycin. TS603 cells (harbouring endogenous R132H IDH1) and TS667 cells (expressing WT IDH1) have been previously described^44^, and were grown in Neurobasal-A media (Invitrogen) supplemented with B27 Supplement (Invitrogen), N2 Supplement (Invitrogen), 20ngml^−1^ epidermal growth factor (Peprotech), 20ngml^−1^ fibroblast growth factor (Peprotech), 20ngml^−1^ platelet-derived growth factor AA (Peprotech), and 100 units ml^−1^ penicillin/streptomycin. BT142 cells harbouring endogenous R132H were obtained from and authenticated by ATCC, and were grown in Neurobasal-A media (Invitrogen) supplemented with B27 Supplement (Invitrogen), N2 Supplement (Invitrogen), 20ngml^−1^ epidermal growth factor (Peprotech), 20 ng ml^−1^ fibroblast growth factor (Peprotech), 20 ng ml^−1^ platelet-derived growth factor AA (Peprotech), and 100 units ml^−1^ penicillin/streptomycin. All primary cells and cell lines were authenticated by ATCC (where applicable), or by analysis of morphological and phenotypic characteristics as well as gene and protein expression.

No cell lines used in this study were found in the database of commonly misidentified cell lines that is maintained by ICLAC and NCBI Biosample. All primary cells and cell lines were tested for mycoplasma contamination using a commercially available kit (PCR-Mycoplasma Test Kit I/C (Promokine PK-CA91-1024), according to the manufacturer's instructions, at the onset of the work (tested negative) and have never exhibited contamination symptoms after initial testing. Derivative cells were transduced *in vitro* with a lentiviral vector-encoding firefly Luciferase and either eGFP (U87 lines) or mCherry (primary GBM lines) fluorescent proteins for combined bioluminescent imaging and flow sorting. For stable shRNA cell lines harbouring isopropyl β-d-1-thiogalactopyranoside (IPTG)-inducible transgenes, expression was induced with 100 μM IPTG five days before experimentation. For polyacrylamide hydrogel studies, cells were plated on fibronectin-conjugated gels for either 8 h (protein analysis) or 24 h (mRNA analysis) prior to harvesting cell lysates.

### Quantitative real-time PCR and *in situ* hybridization

Total cellular RNA was isolated using the mirVana miRNA isolation kit (Life Technologies AM1560), according to the manufacturer's instructions. Reverse transcription was performed from 2 μg of total RNA using random primers (Amersham Biosciences). PCR reactions were performed in triplicate with LightCycler Fast Start DNA Master SYBR Green Mix (Roche) using a Realplex^29,30^ epGradient S Mastercycler (Eppendorf) and the relative amount of cDNA was calculated by the comparative Ct method using the RPS20 ribosomal protein as a control. Primer sequences are: human TNC forward primer 5′-TCTCAGGGTCATTCACCACA-3′, human TNC reverse primer 5′-CA CCGTGCGTGTAATTTCTG-3′, human HIF1α forward primer 5′-CAGTCGACA CAGCCTGGATA-3′, human HIF1α reverse primer 5′-TGTCCTGTGGTGACTT GTCC-3′, SLC2A1 forward primer 5′-GGCATCAACGCTGTCTTCTA-3′, SLC2A1 R 5′-CAGCGAGGCCTATGAGGTG-3′, RPS20 forward primer 5′-AGGACCAGT TCGAATGCCTA-3′, and RPS20 reverse primer 5′-GATTCGATCAACTCAACTC CTG-3′.

For miRNA qPCR analysis, reverse transcription of specific miRNAs (from 10 ng of total RNA) was carried out using the real-time loop primers for each type of miRNA and the TaqMan miRNA RT Kit from Applied Biosystems (Life Technologies 4366596), according to the instructions. cDNA obtained from this step was used to carry out real-time quantitative TaqMan PCR for miR-203 (Life Technologies 4427975, Assay 000507) using the real-time primers provided, according to the instructions. Ct values were converted to fold expression changes (2 —ΔΔCt values) following normalization to U6 small nuclear RNA (Life Technologies 4427975, Assay 001973) or SNORD48 (Life Technologies 4427975, Assay 001006).

miRNA *in situ* hybridization was performed as previously described using formalin-fixed, paraffin-embedded tissue sections (5 μm thickness)^45^. 5′-DIG labelled probes targeting miR-203 (Exiqon 35029-01), U6 small nuclear RNA (positive control, Exiqon 99002-01) or Scramble-miR (negative control, Exiqon 99004-01) were diluted to 40 nM in hybridization buffer. RNA was hybridized overnight at 58 °C for all probes.

### Chromatin immunoprecipitation (ChIP) studies

Samples were cultured under either normoxia or hypoxia (1% oxygen) and ChIP was performed using the ChIP-IT Express kit (Active Motif 5300), according to the instructions. Primer pairs are: intronic TNC set 1 (90 bp) forward primer 5′-AAAGCAACCGTAGGATGACC-3 and reverse primer 5′-ATCCCCACAGTTATTATTGTT-3′, intronic TNC set 2 (149 bp) forward primer 5′-GGCTGTGATTTCTACACAAATGG-3′ and reverse primer 5′-AGGGAATACACCTGGGAAGTG-3′, intronic TNC set 3 (152 bp) forward primer 5′-CTGGTCCCATTTCCAGCTT-3′ and reverse primer 5′-CGA CCCCGAGTAGCTGTTAG-3′, exonic TNC set 4 (142 bp) forward primer 5′-ACA GCCTGCCTACTGTCACC-3′ and reverse primer 5′-GGAAGAAGTACCTGGAG TGTGG-3′, intronic TNC negative control (149 bp) forward primer 5′-GTGAAAT TCAAAATTAAGTTCAACAA-3′ and reverse primer 5′-CAAGTCGCATCCACT CTTGA-3′, and GAPDH positive control (149 bp) forward primer 5′-TACTAGCG GTTTTACGGGCG-3′ and reverse primer 5′-AGAGCGAAGCGGGAGGCT-3′.

### Atomic force microscopy measurements

Atomic force microscopy (AFM) and analysis were performed using an MFP3D-BIO inverted optical atomic force microscope mounted on a Nikon TE2000-U inverted fluorescent microscope (Asylum Research) as described previously (Supplementary Fig. 1a)^11^. Briefly, for pathologist-identified, regionally matched correlations between ECM stiffness and mechanosignalling, patient tissues were immunostained with multiple specific, patient-representative regions (4–10 regions per tumour) identified (with the *x*-and *y*-coordinates noted and images acquired) for further AFM testing in serial sections (Supplementary Fig. 6c left). After patient tumour sections were secured for mechanical testing, the identified testing region was located via *x*- and *y*-coordinates using the optical microscope (Supplementary Fig. 6c right). Individual patient data were combined and graphed to depict correlations and the patient variability within the cohort(s) (Supplementary Fig. 6d,e).

### Nanostring nCounter gene expression signature assessment

NanoString nCounter gene expression analysis^12^ of human GBM tissue biopsies was used to assess the 9-gene expression signature used as a surrogate for tumour aggressiveness^13^. Derived from a 38-gene predictor (validated as a significant predictor of overall survival), the top-ranked 9 genes were selected on the basis of the significance of survival association (fold change level) and technical considerations (including abundance and consistency of amplification of the target gene in FFPE samples)^13^. Aggressiveness reflects the overall survival prediction based on the meta-score calculated with the 9-gene signature^13^.

### Lentivirus-mediated ectopic expression or knockdown

For ectopic expression or knockdown studies, lentiviral vectors (pLV lacI NeoR; gift from J. Lakins, UCSF, USA) were custom built to facilitate cloning of shRNA under inducible IPTG control. Briefly, oligonucleotides corresponding to validated shRNA from The RNAi Consortium (TRC) were designed and annealed to yield duplexes with 5′ and 3′ overhangs complementary to AgeI and EcoRI sites of the modified U6 promoter containing three lacO binding sites for the bacterial repressor protein lacI (Sigma). pLV lacI_NeoR also provides for constitutive nuclear expression via the human EEF1a promoter of lacI tagged at the N terminus with three tandem repeats of the 9E10 myc epitope and at the C terminus with a nuclear localization sequence from SV40 large T antigen. The latter is co-expressed with the neomycin phosphotransferase gene from a bicistronic mRNA via an internal ribosome entry site (IRES) to provide selection of transduced cells with G418. Utilized TNC shRNA was TRCN0000230788.

### Preparation of polyacrylamide gels substrates

Glass and silicon substrates were prepared by glutaraldehyde activation followed by conjugation with 10μg ml^−1^ (glass) or 20 μg ml^−1^ (silicon) fibronectin as described previously^29^. Polyacrylamide hydrogel substrates (soft: 2.5% acrylamide, 0.03% Bis-acrylamide; stiff: 10% acrylamide, 0.5% Bis-acrylamide) were prepared as previously described with one modification: functionalization with succinimidyl ester was with 0.01% N6, 0.01% Bis-acrylamide, 0.025% Irgacure 2959, and 0.002% di(trimethylolpropane) tetraacrylate (Sigma)^46^. Following functionalization with succinimidyl ester, hydrogels were conjugated overnight with 20 μg ml^−1^ fibronectin at 4 °C and rinsed twice with PBS and DMEM before cell plating.

### Immunofluorescence and imaging

Samples were fixed and labelled as previously described^29^, with multiple independent fields imaged on a Zeiss LSM 510 microscope system with either a 20 × Apo NA0.75 air or 40 × ApoLWDNA 1.15 water objective and 488 nm argon, 543 nm HeNe, and 633 nm HeNe excitation lines.

### Clinical data analysis

Previously published and reanalysed microarray data were obtained from the TCGA Research Network (http://cancergenome.nih.gov) and downloaded from the CBio Portal for Cancer Genomics (http://www.cbioportal.org/public-portal/index.do) under the Low Grade Glioma^1^ and the Glioblastoma Multiforme^17–19^ data sets, with mean normalized expression scores (*z* scores) for genes of interest determined. Statistical significance of differences was determined using a two-tailed Mann–Whitney test, with *P* < 0.05 considered to be significant.

Genes used in the hypoxia gene signature include: *ANXA2*, *BTG1*, *CA9*, *EDN1*, *EGR1*, *HMOX1*, *IER3*, *LDHA*, *LGAL3*, *LOXL2*, *PDK1*, *SLC2A1*, *TFRC*, *VDAC1* and *VEGFA*.

### Statistics and reproducibility

All quantitative results were assessed by unpaired Student's *t*-test after confirming that the data met appropriate assumptions (normality, homogeneous variance, and independent sampling), non-parametric Wilcox/Mann–Whitney exact test (using the normal approximation for the U score), or the Kolmogorov–Smirnov distribution test (α = 0.05), all two-tailed. Unless otherwise indicated, all data were plotted with standard deviation error bars, and variances between groups being statistically compared are similar. For AFM mechanical testing of clinical specimens, multiple regions were assessed (10 regions per patient), and regions were pooled per patient to establish a mean Young's modulus per patient; statistical analysis was performed on patient means (unless otherwise noted). Animal cohort and *in vitro* sample sizes were chosen to provide 85% power to detect an effect size of 2.5 with a two-sided error of less than or equal to 5%. All results were reproduced at least twice in the laboratory and the n-value denotes the sample size used in statistical testing. Graphpad/Prism software was used to conduct the statistical analysis of the data. *P* values less than or equal to 0.05 were considered to be significant. For animal studies, animals were randomly distributed among the different conditions by the investigator since the animals did not exhibit any size or appearance differences at the onset of the experiments. No animals were excluded. For mouse and clinical studies, mechanical testing was performed blinded and immunostaining intensity of tissue sections was scored blinded.

### Data availability

Previously published microarray data^1,17,18^ that were reanalysed here are available from the TCGA Research Network (http://cancergenome.nih.gov) via download from the CBio Portal for Cancer Genomics (http://www.cbioportal.org/public-portal/index.do) under the Low Grade Glioma^1^ and the Glioblastoma Multiforme^17–19^ data sets. All other relevant data that support the findings and conclusions of this study are available from the corresponding author on request.

## Supplementary Material

**Supplementary Figure 1** Cellularity, fibrillar collagen and vascularity are independent of ECM stiffness. a, Scatter plots of ECM stiffness measured by AFM in a human patient sample analyzed immediately post resection from the operating room (Fresh), post snap freezing and analyzed either within 30 minutes of being thawed in a cocktail of protein inhibitors (Frozen) or treated with either PBS (PBS) or hyaluronidase (HAse) for 60 minutes (6 samples/group were tested for all groups with the exception of the “HAse 60 mins” group where 5 samples were assessed). All samples were pooled per group with 5-6 regions tested per sample (pooled group means indicated with red lines). Group means are indicated in red (6 samples/ group were tested for all groups with the exception of the “HAse 60 mins” group where 5 samples were assessed). b, Immunohistochemistry images of patient tissue immunostained for neuronal (SMI31) or astrocytic (GFAP) processes indicating a switch in the type but not density of the cellular processes in normal versus GBM brains. Scale Bar 70μm. c, Correlation between measured ECM stiffness and cell number in the analyzed area revealing no relationship between the two variables (linear and exponential regressions, n=100 areas (10 patient biopsies with 10 areas/patient), insert: representative image of the AFM method for tissue analysis). Scale Bar 150μm. d, Second harmonic generation (SHG) image of fibrillary collagen (purple) and CD31-immunostained vasculature (red) in human GBMs (insert: composite image of SHG and CD31 in a normal human brain). Scale Bar 70μm. e, Immunofluorescence images of astrocytic processes (GFAP, red) and vasculature (Collagen 1, green) in human gliotic (top) and GBM (bottom) biopsies. Scale Bar 50μm. f, Correlation between measured ECM stiffness and percent vascularity of the tissue (percent area positive for FVIII stain) revealing no relationship between the two variables (linear and exponentia regression, n=10 patients). g, Distribution of ECM stiffness measured by AFM in two (labeled as mGBM model 1 and mGBM model 2) mouse xenograft models of GBM (n=4 mice/group), normal mouse brain (n=3 mice) as well as mGBM model 2 treated with an antiangiogenic agent, avastin (labeled as mGBM model 2 + B20, n=5 mice) revealing a slight but nonsignificant reduction in ECM stiffness upon B20-mediated normalization of GBM vasculature in mouse models of GBM. All samples were pooled per group with 5 regions tested per sample (pooled group means indicated with yellow lines).
**Supplementary Figure 2** R132H IDH1 expression modifies TNC expression and ECM stiffness. a, Immunofluorescence images and quantifications of WT and R132H IDH1 patient tissues immunostained for DAPI(blue), aggrecan (green), and versican (red) (mean+/- s.e.m., n=7 mice/group, ns by unpaired 2-sided *t*-test). b, Immunofluorescence images of patient tissues immunostained for the lecticans, aggrecan (green) and versican (red). c, Immunoblot analysis confirming the R132H and WT IDH1 status in U87 GBM line. d, Kaplan-Meier graph showing survival of xenograft mice injected orthotopically with either U87 WT IDH1 (blue, n=7 mice) or U87 R132H IDH1 (red, n=7 mice) human GBM cells. e, Histogram showing the distribution of ECM stiffness measured by AFM in xenograft tumors derived from U87 WT (blue) or R132H mutant (red) IDH1 cells (n=5 mice/group WT IDH1, n=4 mice/group R132H IDH1, two-sided Kolmogorov-Smirnov test P=9.7e-4). f, Immunofluorescence mages and quantification of U87 IDH1 WT and R132H mutant xenograft tumors immunostained for pMLC2 (green, top) and pFAK397 (green, bottom) with propidium iodide (PI, red) (mean+/-s.e.m., n=7 mice/group, unpaired 2-sided *t*-test P=0.0007). g, Immunofluorescence images and quantification of TNC (green) with PI (red) in xenograft tumors derived from either WT or R132H IDH1 human primary cells (mean+/-s.e.m., n=7 mice/group, unpaired 2-sided *t*-test P=0.0087). h, Immunoblot analysis confirming lentiviral shRNA construct targeting of TNC (top). immunofluorecence images of cellular morphology (F-actin in green and DAPI in blue) of scramble shRNA and TNC shRNA cells (bottom). Scale Bar 10μm. i, TNC knockdown in mouse xenograft tumors by mRNA (top) and immunofluorescence (bottom) in scramble and TNC shRNA constructs (mean+/-s.d., n= 5 mice/group, unpaired 2-sided *t*-test P=0.014). Scale Bars 50μm, unless otherwise indicated.
**Supplementary Figure 3** HIF1α and miR-203 regulate TNC expression. a, Distribution of ECM stiffness measured by AFM in xenograft tumors arising from primary R132H IDH1 cells in either HIF1α-positive or -negative regions surrounding areas of necrosis (n=4 mice, two-sided Kolmogorov-Smirnov test P=3.7e-2). All samples were pooled per group with 5 regions tested per sample (pooled group means indicated with yellow lines). b, Immunofluorescence images of xenograft tumors derived from cells expressing a control scramble shRNA or an shRNA for TNC shRNA immunostained for HIF1α with PI (red). c, mRNA expression of HIF1α (left) and TNC(right) in WT IDH1 cells expressing either scramble shRNA or shRNA targeting HIF1α, represented as % of shCNL (mean+/-s.e.m., n=6/group, unpaired 2-sided test P=0.001). d, Quantification of HIF1α (left) and TNC(right) protein expression in IDH1 WT cells with and without hypoxia, as indicated (mean+/-s.e.m., n=6/group, one-way ANOVA with Tukey's multiple comparisons test P=0.0013). e, Representative immunoblot of WT and R132H IDH1 primary GBM cells plated on laminin-coated tissue culture plates under normoxic and hypoxic (1% oxygen) conditions probed for TNC and HIF1α. f, Immunoblot of WT IDH1 primary human GBM cells, expressing either scramble control shRNA or shRNA targeting HIF1α, plated on lamin-coated tissue culture plates under normoxic or hypoxic (1% oxygen) conditions, as indicated, and probed for TNC induction. g, immunofluorescence images of xenograft tumors derived from either WT or R132H IDH1 primary human GBM cells immunostained for HIF1α (green) with PI (red); asterisks indicate areas of necrosis. Scale Bars 50μm.
**Supplementary Figure 4** Endogenous and ectopic R132H IDH1 1° GBMs cannot tune HIF1α. a, Immunoblots and quantification of primary human glioma cells harboring an endogenous R132H IDH1 mutation (top: TS603, bottom: BT142) plated on soft (140Pa) or stiff (>6kPa) substrates under normoxic and hypoxic (1% oxygen) conditions and probed for HIF1α and TNC protein expressions, as well as actin (graph shows means of 2 biological replicate samples per group) b, Immunoblots and quantification of WT IDH1 primary human glioma cells (TS667) plated on soft (140Pa) or stiff (>6kPa) substrates under normoxic and hypoxic (1% oxygen) conditions and probed for HIF1α and TNC protein expressions, as well as actin (graph shows means of 2 biological replicate samples per group,) c, Immunoblot of R132H IDH1 primary human GBM cells expressing either vector control or constitutively active HIF1α (CA- HIF1α) plated on soft (140Pa) gels under normoxic and hypoxic (1% oxygen) conditions, as indicated, and probed for HIF1α protein expression, as well as HIF1α -driven protein expressions of TNC and LOXL2.
**Supplementary Figure 5** miR-203 targets the HIF1α and TNC 3′ UTRs. a, Diagram of miR-203 targeting seed sequences located in 3′-untranslated regions (3′ UTRs) of human HIF1α and TNC mRNAs. b, Quantification of miR-203 expression after RNA immunoprecipitation (RIP) using AGO2 as a bait validates that antagomiR-mediated targeting of miR-203 reduces miR-203 loading into the RISC (graph shows mean of 2 IP lysates per group). c, Immunoblots for TNC, HIF1α, LOXL2 (as HIF1α target gene) and actin from primary human GBM cells expressing R132H IDH1 (+/-an antagomiR control or an antagomiR targeting miR-203, +/- 1% hypoxia). d, Quantification of HIF1α and TNC protein expressions in primary human GBM cells expressing R132H IDH1 (+/-an antagomiR control or an antagomiR targeting miR-203, +/- 1% hypoxia) (means+/-s.e.m., n=3 lysates/group, One-way ANOVA with Tukey's multiple comparisons test P=0.0011).
**Supplementary Figure 6** Regional assessment of ECM stiffness and mechanosignaling. a, Maps of ECM stiffness are obtained using an AFM tip where a 5μm diameter bead is attached to the end of a cantilever. The AFM tip was brought down to indent the sample, during which the motion of the z-piezo and the applied force were recorded. The ECM stiffness was computed from the displacement of the z-piezo minus the bending of the cantilever. The number of ECM stiffness measurements made was operator-determined by the x- and y-step sizes input for a 90μm by 90μm grid. The example shown demonstrates how step sizes of 14 (in the x-and y-directions) result in 196 independent mechanical measurements. b, For regionally-matched correlations between ECM stiffness and mechanosignaling, patient (and xenograft) tissues were immunostained for TNC (green) and pFAK (red). Multiple specific, patient-representative regions (4-10 regions per tumor) were identified (with the x- and y-coordinates noted and images acquired) for further AFM testing in a serial section. For subsequent AFM testing, after patient (and xenograft) tumor sections were secured for testing, the identified testing region was located via x- and y-coordinates using the optical microscope. c, Left: Immunohistochemistry mages of patient tumors immunostained for TNC (green) and pFAK (red) with DAPI (blue) with regions for AFM analysis in serial sections denoted by white boxes. Graphs illustrate individual patient correlations between TNC and pFAK. Right: AFM maps of patient tumors. Graphs illustrate individual patient correlations between elastic modulus (stiffness) and pFAK. d, The data underlying (c left) was combined into graphs depicting regiona matching of ECM stiffness and pFAK (left) and TNC and pFAK (right) to illustrate not only the correlations between the variables, but also the variability between patients within the cohort (n=3 patient samples/group, One-way ANOVA with Tukey's comparison P=0.0011). e, The data underlying (c right) was combined into a graph depicting both the patient-specific trends in elastic modulus (stiffness) and the patient variability within the cohort (n=10 patients, 4 regions/patient sample, linear regression analysis yielding p<0.0001 for both plots with R2=0.4812 for pFAK:stiffness data and R2=0.5850 for pFAK:TNC data). Scale Bars 200μm (left panels) and 20μm (right panels), as indicated.
**Supplementary Figure 7** Full Scans of Key Blots

## References

[1] Brat DJ (2015). Comprehensive, integrative genomic analysis of diffuse lower-grade gliomas. N Engl J Med.

[2] Yan H (2009). IDH1 and IDH2 mutations in gliomas. N Engl J Med.

[3] Van den Bent MJ (2013). Adjuvant procarbazine, lomustine, and vincristine chemotherapy in newly diagnosed anaplastic oligodendroglioma: long-term follow-up of EORTC brain tumor group study 26951. J Clin Oncol.

[4] Cairncross G (2013). Phase III trial of chemoradiotherapy for anaplastic oligodendroglioma: long-term results of RTOG 9402. J Clin Oncol.

[5] Losman JAJA, Kaelin WG (2013). What a difference a hydroxyl makes: mutant IDH, (R)-2-hydroxyglutarate, and cancer. Genes Dev.

[6] Zhao S (2009). Glioma-derived mutations in IDH1 dominantly inhibit IDH1 catalytic activity and induce HIF-1. Science.

[7] Koivunen P (2012). Transformation by the (R)-enantiomer of 2-hydroxyglutarate linked to EGLN activation. Nature.

[8] Levental KR (2009). Matrix crosslinking forces tumor progression by enhancing integrin signaling. Cell.

[9] Mouw JK (2014). Tissue mechanics modulate microRNA-dependent PTEN expression to regulate malignant progression. Nat Med.

[10] Kumar S, Weaver VM (2009). Mechanics, malignancy, and metastasis: the force journey of a tumor cell. Cancer Metastasis Rev.

[11] Lopez JI, Kang I, You WK, McDonald DM, Weaver VM (2011). *In situ* force mapping of mammary gland transformation. Integr Biol (Camb).

[12] Geiss GK (2008). Direct multiplexed measurement of gene expression with color-coded probe pairs. Nat Biotechnol.

[13] Colman H (2010). A multigene predictor of outcome in glioblastoma. Neuro-Oncol.

[14] Ruoslahti E (1996). Brain extracellular matrix. Glycobiology.

[15] Wade A (2013). Proteoglycans and their roles in brain cancer. FEBS J.

[16] Zimmermann DR, Dours-Zimmermann MT (2008). Extracellular matrix of the central nervous system: from neglect to challenge. Histochem Cell Biol.

[17] Cancer Genome Atlas Research Network (2008). Comprehensive genomic characterization defines human glioblastoma genes and core pathways. Nature.

[18] Brennan CW (2013). The somatic genomic landscape of glioblastoma. Cell.

[19] Masson N, Ratcliffe PJ (2014). Hypoxia signaling pathways in cancer metabolism: the importance of co-selecting interconnected physiological pathways. Cancer Metab.

[20] Koperek O, Akin E, Asari R, Niederle B, Neuhold N (2013). Expression of hypoxia-inducible factor 1 α in papillary thyroid carcinoma is associated with desmoplastic stromal reaction and lymph node metastasis. Virchows Arch.

[21] Midwood KS, Orend G (2009). The role of tenascin-C in tissue injury and tumorigenesis. J Cell Commun Signal.

[22] Turcan S (2012). IDH1 mutation is sufficient to establish the glioma hypermethylator phenotype. Nature.

[23] Chesnelong C (2014). Lactate dehydrogenase A silencing in IDH mutant gliomas. Neuro-Oncol.

[24] Tarhonskaya H (2014). Non-enzymatic chemistry enables 2-hydroxyglutarate-mediated activation of 2-oxoglutarate oxygenases. Nat Commun.

[25] Wierenga ATJ, Vellenga E, Schuringa JJ (2014). Convergence of hypoxia and TGFβ pathways on cell cycle regulation in human hematopoietic stem/progenitor cells. PLoS ONE.

[26] Marucci G (2015). Pathological spectrum in recurrences of glioblastoma multiforme. Pathologica.

[27] Li R (2015). Genetic and clinical characteristics of primary and secondary glioblastoma is associated with differential molecular subtype distribution. Oncotarget.

[28] Barcellos-Hoff MH, Ravani SA (2000). Irradiated mammary gland stroma promotes the expression of tumorigenic potential by unirradiated epithelial cells. Cancer Res.

[29] Paszek MJ (2005). Tensional homeostasis and the malignant phenotype. Cancer Cell.

[30] Miroshnikova YA (2011). Engineering strategies to recapitulate epithelial morphogenesis within synthetic three-dimensional extracellular matrix with tunable mechanical properties. Phys Biol.

[31] Chappell WH (2011). Ras/Raf/MEK/ERK and PI3K/PTEN/Akt/mTOR inhibitors: rationale and importance to inhibiting these pathways in human health. Oncotarget.

[32] Chang JH (2016). MicroRNA-203 modulates the radiation sensitivity of human malignant glioma cells. Int J Radiat Oncol Biol Phys.

[33] Liao H (2015). MiR-203 downregulation is responsible for chemoresistance in human glioblastoma by promoting epithelial-mesenchymal transition via SNAI2. Oncotarget.

[34] Le LTN (2016). Loss of miR-203 regulates cell shape and matrix adhesion through ROBO1/Rac/FAK in response to stiffness. J Cell Biol.

[35] Scherr M (2007). Lentivirus-mediated antagomir expression for specific inhibition of miRNA function. Nucleic Acids Res.

[36] Williams SC (2011). R132H-mutation of isocitrate dehydrogenase-1 is not sufficient for HIF-1α upregulation in adult glioma. Acta Neuropathol.

[37] Xu W (2011). Oncometabolite 2-hydroxyglutarate is a competitive inhibitor of α-ketoglutarate-dependent dioxygenases. Cancer Cell.

[38] Noushmehr H (2010). Identification of a CpG island methylator phenotype that defines a distinct subgroup of glioma. Cancer Cell.

[39] Evans SM (2004). Hypoxia is important in the biology and aggression of human glial brain tumors. Clin Cancer Res.

[40] Vordermark D (2004). Significance of hypoxia in malignant glioma. Re: Evans et al. Hypoxia is important in the biology and aggression of human glial brain tumors. Clin Cancer Res.

[41] Lu KV (2012). VEGF inhibits tumor cell invasion and mesenchymal transition through a MET/VEGFR2 complex. Cancer Cell.

[42] Johnson KR, Leight JL, Weaver VM (2007). Demystifying the effects of a three-dimensional microenvironment in tissue morphogenesis. Methods Cell Biol.

[43] Sarkaria JN (2006). Use of an orthotopic xenograft model for assessing the effect of epidermal growth factor receptor amplification on glioblastoma radiation response. Clin Cancer Res.

[44] Turcan S (2013). Efficient induction of differentiation and growth inhibition in IDH1 mutant glioma cells by the DNMT inhibitor decitabine. Oncotarget.

[45] Kriegel AJ, Liang M (2013). MicroRNA *in situ* hybridization for formalin fixed kidney tissues. J Vis Exp.

[46] Lakins JN, Chin AR, Weaver VM (2012). Exploring the link between human embryonic stem cell organization and fate using tension-calibrated extracellular matrix functionalized polyacrylamide gels. Methods Mol Biol.

