# Supplementary material for: Tissue mechanics promote IDH1-dependent HIF1α–tenascin C feedback to regulate glioblastoma aggression
Source: Nat Cell Biol. Author manuscript; Available in PMC 2017 Dec 1. (PMC5361403; doi:10.1038/ncb3429)
Supplement: Supplementary file 1 — Supplementary Figure 1 Cellularity, fibrillar collagen and vascularity are independent of ECM stiffness. a, Scatter plots of ECM stiffness measured by AFM in a human patient sample analyzed immediately post resection from the operating room (Fresh), post snap freezing and analyzed either within 30 minutes of being thawed in a cocktail of protein inhibitors (Frozen) or treated with either PBS (PBS) or hyaluronidase (HAse) for 60 minutes (6 samples/group were tested for all groups with the exception of the “HAse 60 mins” group where 5 samples were assessed). All samples were pooled per group with 5-6 regions tested per sample (pooled group means indicated with red lines). Group means are indicated in red (6 samples/ group were tested for all groups with the exception of the “HAse 60 mins” group where 5 samples were assessed). b, Immunohistochemistry images of patient tissue immunostained for neuronal (SMI31) or astrocytic (GFAP) processes indicating a switch in the type but not density of the cellular processes in normal versus GBM brains. Scale Bar 70μm. c, Correlation between measured ECM stiffness and cell number in the analyzed area revealing no relationship between the two variables (linear and exponential regressions, n=100 areas (10 patient biopsies with 10 areas/patient), insert: representative image of the AFM method for tissue analysis). Scale Bar 150μm. d, Second harmonic generation (SHG) image of fibrillary collagen (purple) and CD31-immunostained vasculature (red) in human GBMs (insert: composite image of SHG and CD31 in a normal human brain). Scale Bar 70μm. e, Immunofluorescence images of astrocytic processes (GFAP, red) and vasculature (Collagen 1, green) in human gliotic (top) and GBM (bottom) biopsies. Scale Bar 50μm. f, Correlation between measured ECM stiffness and percent vascularity of the tissue (percent area positive for FVIII stain) revealing no relationship between the two variables (linear and exponentia regression, n=10 patients). g, Distri [file NIHMS852654-supplement-supplement_1.pdf]

nature cell biology

DOI: 10.1038/ncb3429

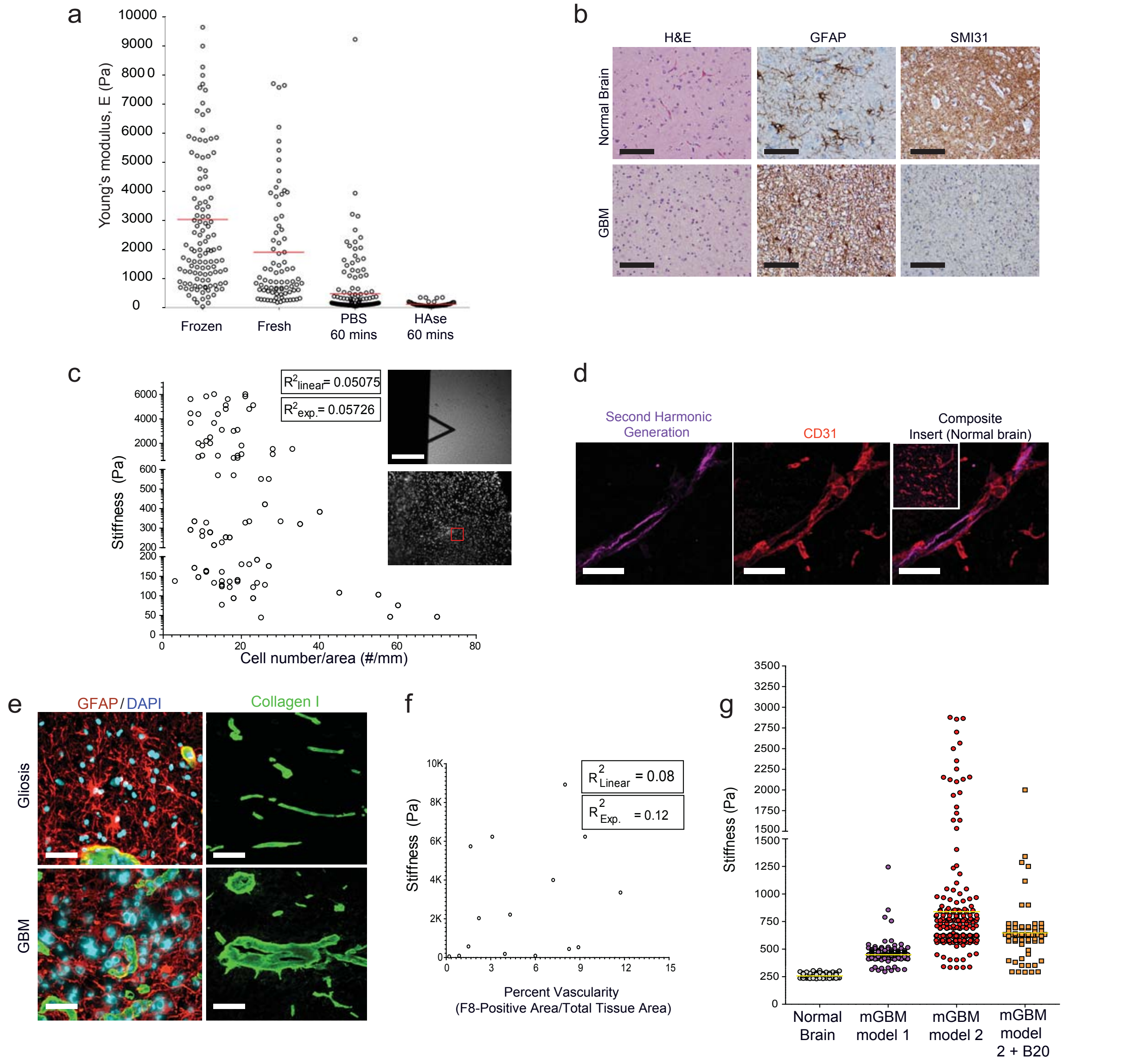

Supplementary Figure 1 Cellularity, fibrillar collagen and vascularity are independent of ECM stiffness. a, Scatter plots of ECM stiffness measured by AFM in a human patient sample analyzed immediately post resection from the operating room (Fresh), post snap freezing and analyzed either within 30 minutes of being thawed in a cocktail of protein inhibitors (Frozen) or treated with either PBS (PBS) or hyaluronidase (HAse) for 60 minutes (6 samples/group were tested for all groups with the exception of the "HAse 60 mins" group where 5 samples were assessed). All samples were pooled per group with 5-6 regions tested per sample (pooled group means indicated with red lines). Group means are indicated in red (6 samples/group were tested for all groups with the exception of the "HAse 60 mins" group where 5 samples were assessed). b, Immunohistochemistry images of patient tissue immunostained for neuronal (SMI31) or astrocytic (GFAP) processes indicating a switch in the type but not density of the cellular processes in normal versus GBM brains. Scale Bar 70μm. c, Correlation between measured ECM stiffness and cell number in the analyzed area revealing no relationship between the two variables (linear and exponential regressions, n=100 areas (10 patient biopsies with 10 areas/patient), insert: representative image of the AFM method for tissue analysis). Scale Bar 150μm. d, Second harmonic generation (SHG) image of fibrillary collagen (purple) and CD31-immunostained vasculature (red) in human GBMs (insert: composite image of SHG and CD31 in a normal human brain). Scale Bar 70μm. e, Immunofluorescence images of astrocytic processes (GFAP, red) and vasculature (Collagen 1, green) in human gliotic (top) and GBM (bottom) biopsies. Scale Bar 50μm. f, Correlation between measured ECM stiffness and percent vascularity of the tissue (percent area positive for FVIII stain) revealing no relationship between the two variables (linear and exponential regression, n=10 patients). g, Distribution of ECM stiffness measured by AFM in two (labeled as mGBM model 1 and mGBM model 2) mouse xenograft models of GBM (n=4 mice/group), normal mouse brain (n=3 mice) as well as mGBM model 2 treated with an antiangiogenic agent, avastin (labeled as mGBM model 2 + B20, n=5 mice) revealing a slight but non-significant reduction in ECM stiffness upon B20-mediated normalization of GBM vasculature in mouse models of GBM. All samples were pooled per group with 5 regions tested per sample (pooled group means indicated with yellow lines).

© 2016 Macmillan Publishers Limited, part of Springer Nature. All rights reserved.

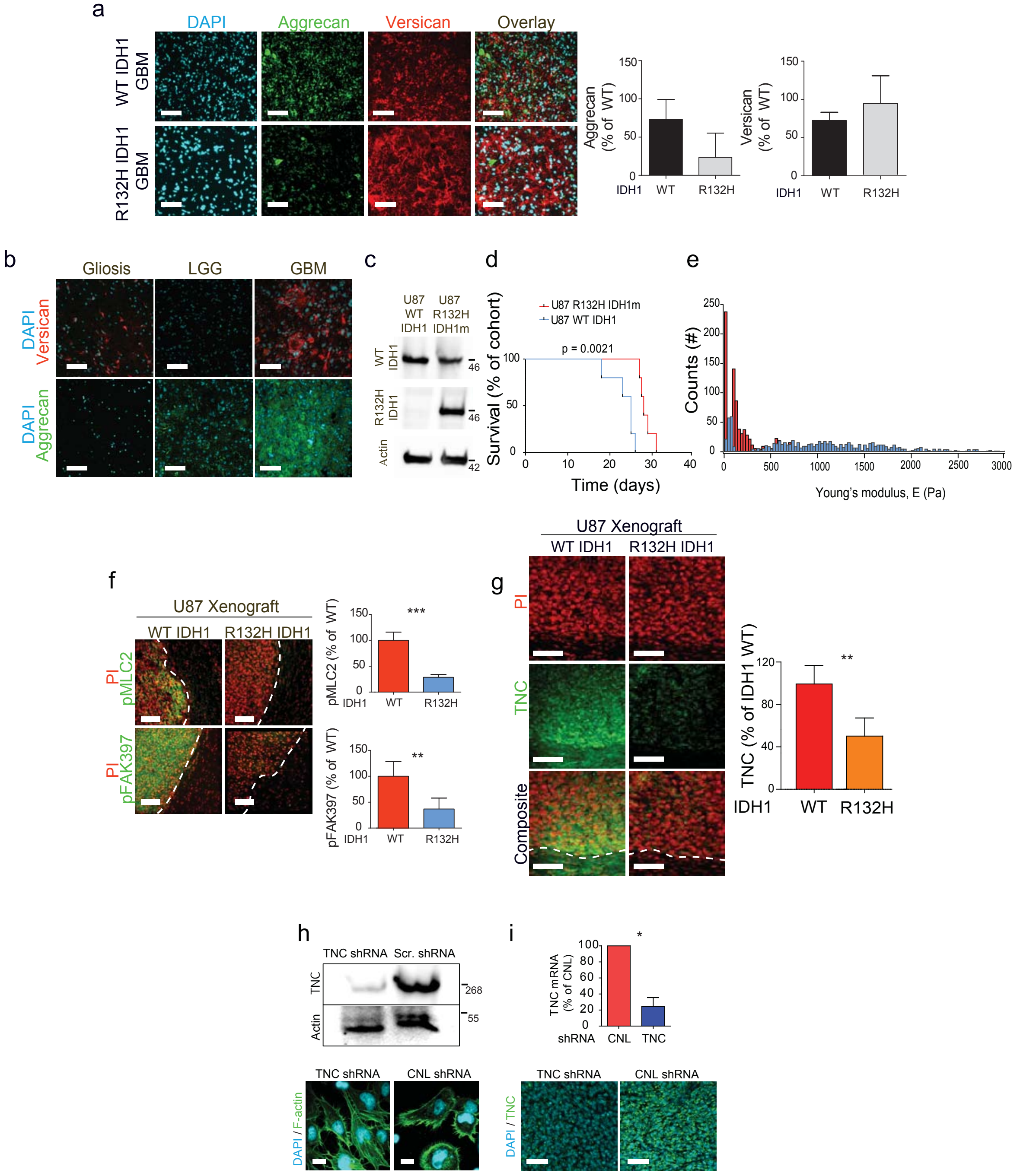

Supplementary Figure 2 R132H IDH1 expression modifies TNC expression and ECM stiffness. a, Immunofluorescence images and quantifications of WT and R132H IDH1 patient tissues immunostained for DAPI(blue), aggrecan (green), and versican (red) (mean+/- s.e.m., n=7 mice/group, ns by unpaired 2-sided t-test). b, Immunofluorescence images of patient tissues immunostained for the lecticans, aggrecan (green) and versican (red). c, Immunoblot analysis confirming the R132H and WT IDH1 status in U87 GBM line. d, Kaplan-Meier graph showing survival of xenograft mice injected orthotopically with either U87 WT IDH1 (blue, n=7 mice) or U87 R132H IDH1 (red, n=7 mice) human GBM cells. e, Histogram showing the distribution of ECM stiffness measured by AFM in xenograft tumors derived from U87 WT (blue) or R132H mutant (red) IDH1 cells (n=5 mice/group WT IDH1, n=4 mice/group R132H IDH1, two-sided Kolmogorov-Smirnov test P=9.7e-4). f, Immunofluorescence images and quantification of U87 IDH1 WT and R132H mutant xenograft tumors immunostained for pMLC2 (green, top) and pFAK397 (green, bottom) with propidium iodide (PI, red) (mean+/-s.e.m., n=7 mice/group, unpaired 2-sided t-test P=0.0007). g, Immunofluorescence images and quantification of TNC (green) with PI (red) in xenograft tumors derived from either WT or R132H IDH1 human primary cells (mean+/-s.e.m., n=7 mice/group, unpaired 2-sided t-test P=0.0087). h, Immunoblot analysis confirming lentiviral shRNA construct targeting of TNC (top). Immunofluorecence images of cellular morphology (F-actin in green and DAPI in blue) of scramble shRNA and TNC shRNA cells (bottom). Scale Bar 10μm. i, TNC knockdown in mouse xenograft tumors by mRNA (top) and immunofluorescence (bottom) in scramble and TNC shRNA constructs (mean+/-s.d., n= 5 mice/group, unpaired 2-sided t-test P=0.014). Scale Bars 50μm, unless otherwise indicated.

© 2016 Macmillan Publishers Limited, part of Springer Nature. All rights reserved.

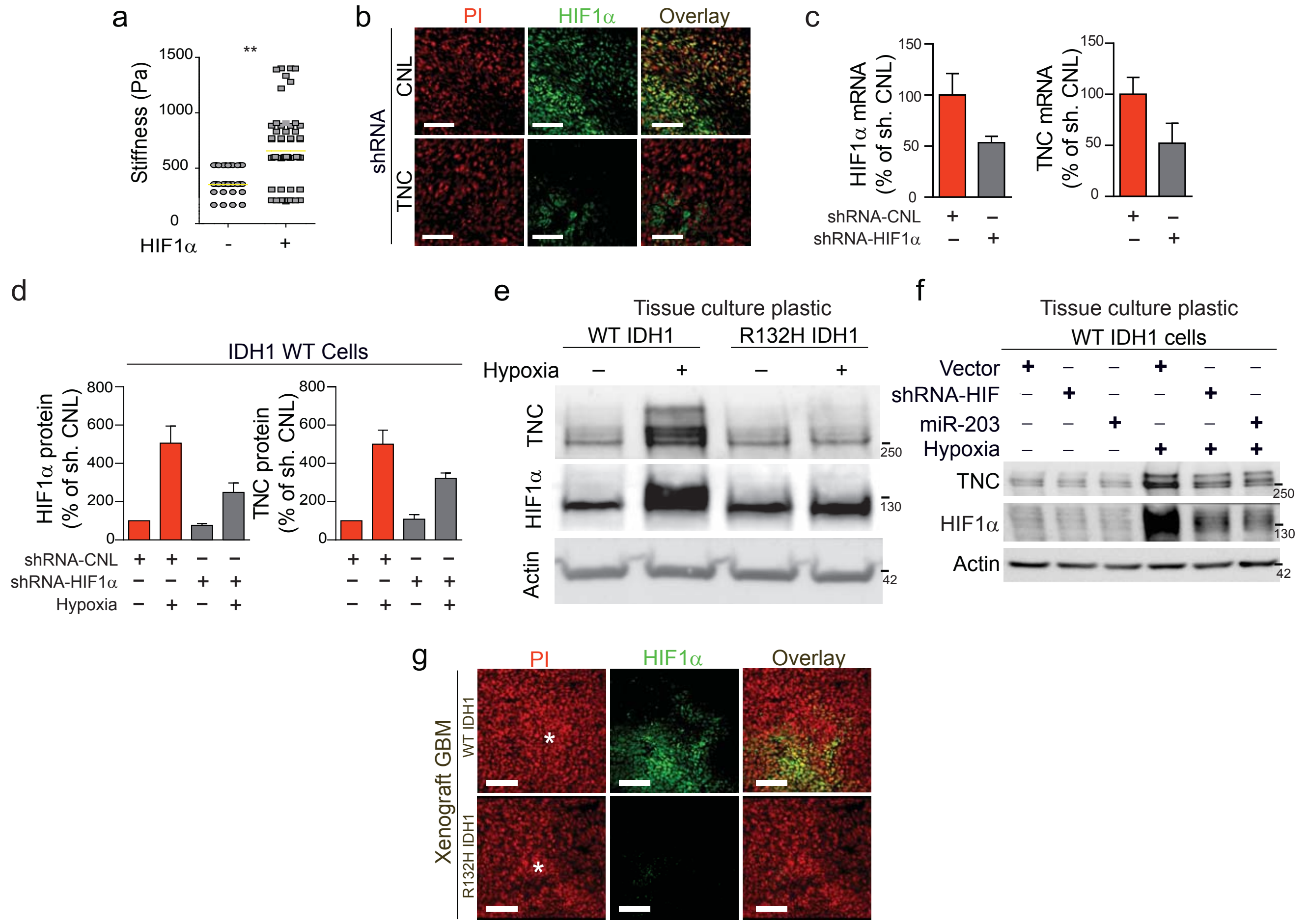

Supplementary Figure 3 HIF1α and miR-203 regulate TNC expression. a, Distribution of ECM stiffness measured by AFM in xenograft tumors arising from primary R132H IDH1 cells in either HIF1α-positive or -negative regions surrounding areas of necrosis (n=4 mice, two-sided Kolmogorov-Smirnov test P=3.7e-2). All samples were pooled per group with 5 regions tested per sample (pooled group means indicated with yellow lines). b, Immunofluorescence images of xenograft tumors derived from cells expressing a control scramble shRNA or an shRNA for TNC shRNA immunostained for HIF1α with PI (red). c, mRNA expression of HIF1α (left) and TNC(right) in WT IDH1 cells expressing either scramble shRNA or shRNA targeting HIF1α, represented as % of shCNL (mean+/-s.e.m., n=6/group, unpaired 2-sided test P=0.001). d, Quantification of HIF1α (left) and TNC(right) protein expression in IDH1 WT cells with and without hypoxia, as indicated (mean+/-s.e.m., n=6/group, one-way ANOVA with Tukey's multiple comparisons test P=0.0013). e, Representative immunoblot of WT and R132H IDH1 primary GBM cells plated on laminin-coated tissue culture plates under normoxic and hypoxic (1% oxygen) conditions probed for TNC and HIF1α. f, Immunoblot of WT IDH1 primary human GBM cells, expressing either scramble control shRNA or shRNA targeting HIF1α, plated on lamin-coated tissue culture plates under normoxic or hypoxic (1% oxygen) conditions, as indicated, and probed for TNC induction. g, Immunofluorescence images of xenograft tumors derived from either WT or R132H IDH1 primary human GBM cells immunostained for HIF1α (green) with PI (red); asterisks indicate areas of necrosis. Scale Bars 50µm.

© 2016 Macmillan Publishers Limited, part of Springer Nature. All rights reserved.

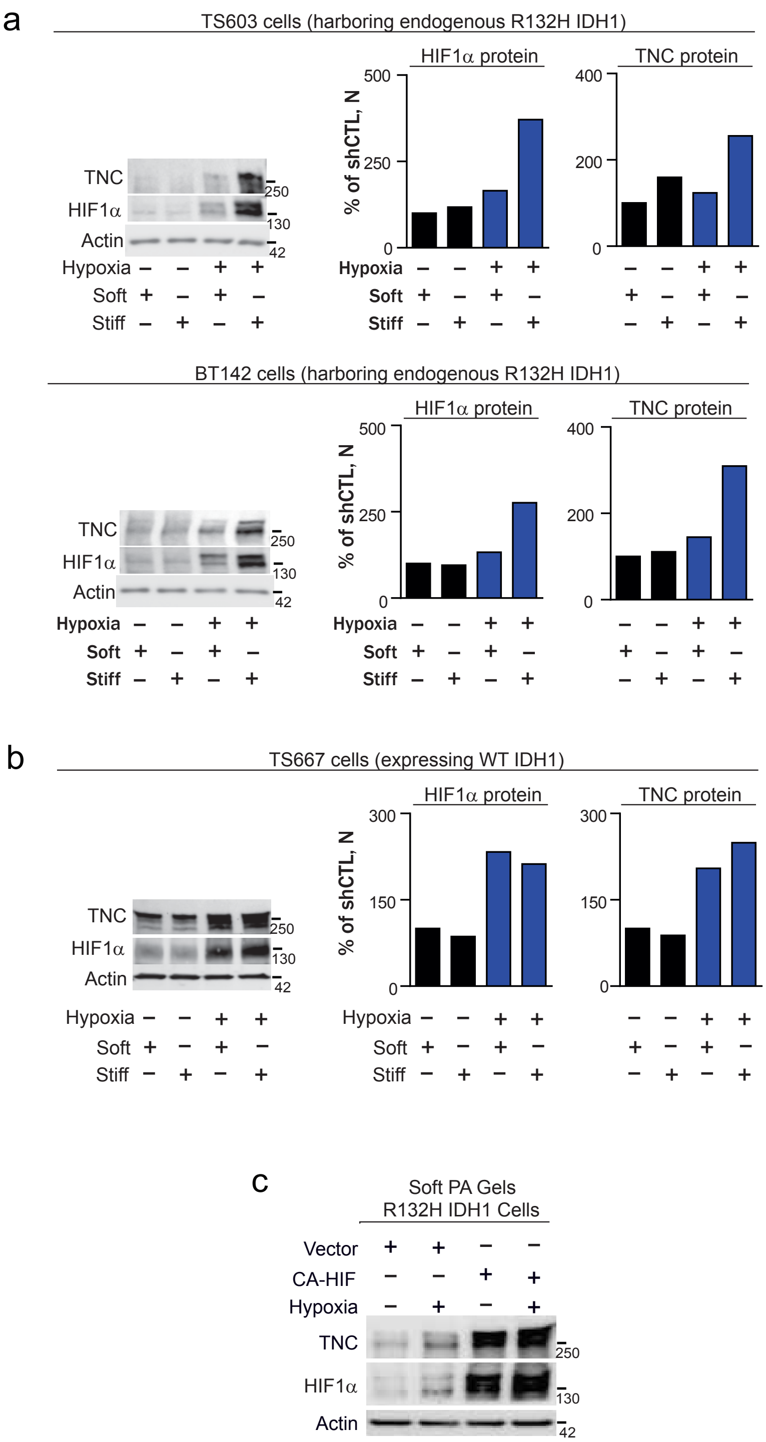

Supplementary Figure 4 Endogenous and ectopic R132H IDH1 1° GBMs cannot tune HIF1α. a, Immunoblots and quantification of primary human glioma cells harboring an endogenous R132H IDH1 mutation (top: TS603, bottom: BT142) plated on soft (140Pa) or stiff (>6kPa) substrates under normoxic and hypoxic (1% oxygen) conditions and probed for HIF1α and TNC protein expressions, as well as actin (graph shows means of 2 biological replicate samples per group) b, Immunoblots and quantification of WT IDH1 primary human glioma cells (TS667) plated on soft (140Pa) or stiff (>6kPa) substrates under normoxic and hypoxic (1% oxygen) conditions and probed for HIF1α and TNC protein expressions, as well as actin (graph shows means of 2 biological replicate samples per group,) c, Immunoblot of R132H IDH1 primary human GBM cells expressing either vector control or constitutively active HIF1α (CA- HIF1α) plated on soft (140Pa) gels under normoxic and hypoxic (1% oxygen) conditions, as indicated, and probed for HIF1α protein expression, as well as HIF1α -driven protein expressions of TNC and LOXL2.

© 2016 Macmillan Publishers Limited, part of Springer Nature. All rights reserved.

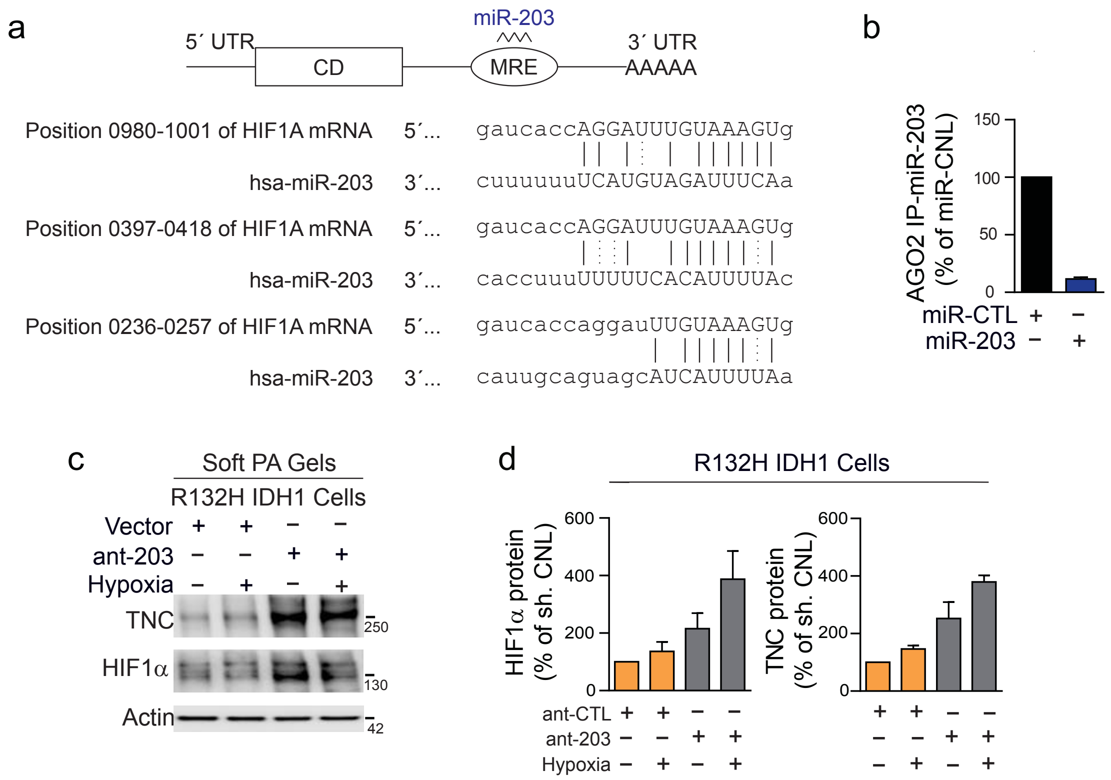

Supplementary Figure 5 miR-203 targets the HIF1α and TNC 3' UTRs. a, Diagram of miR-203 targeting seed sequences located in 3'-untranslated regions (3' UTRs) of human HIF1α and TNC mRNAs. b, Quantification of miR-203 expression after RNA immunoprecipitation (RIP) using AGO2 as a bait validates that antagomiR-mediated targeting of miR-203 reduces miR-203 loading into the RISC (graph shows mean of 2 IP lysates per group). c, Immunoblots for TNC, HIF1α, LOXL2 (as HIF1α target gene) and actin from primary human GBM cells expressing R132H IDH1 (+/-an antagomiR control or an antagomiR targeting miR-203, +/- 1% hypoxia). d, Quantification of HIF1α and TNC protein expressions in primary human GBM cells expressing R132H IDH1 (+/-an antagomiR control or an antagomiR targeting miR-203, +/- 1% hypoxia) (means+/-s.e.m., n=3 lysates/group, One-way ANOVA with Tukey's multiple comparisons test P=0.0011).

© 2016 Macmillan Publishers Limited, part of Springer Nature. All rights reserved.

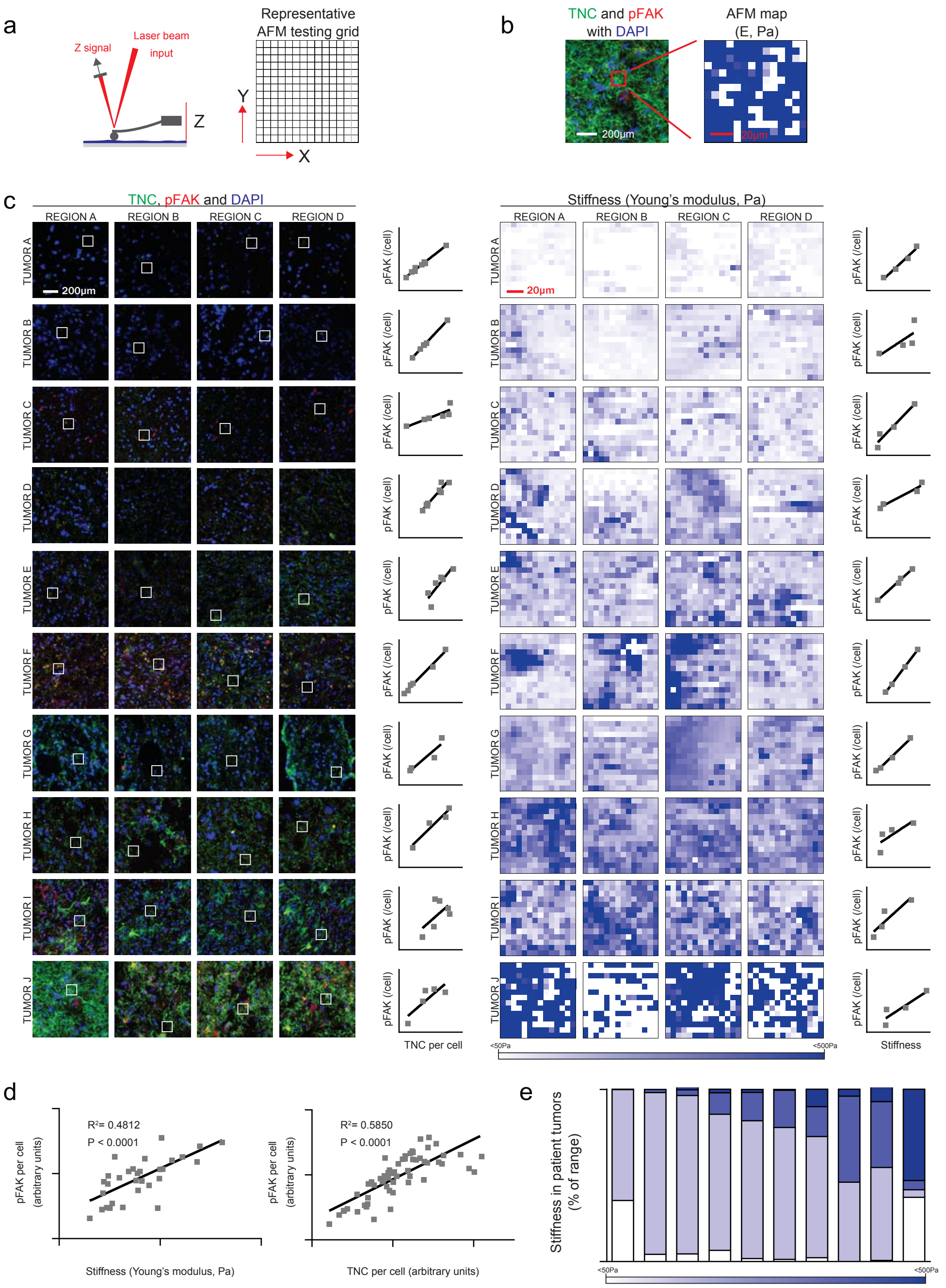

Supplementary Figure 6 Regional assessment of ECM stiffness and mechanosignaling. a, Maps of ECM stiffness are obtained using an AFM tip where a 5µm diameter bead is attached to the end of a cantilever. The AFM tip was brought down to indent the sample, during which the motion of the z-piezo and the applied force were recorded. The ECM stiffness was computed from the displacement of the z-piezo minus the bending of the cantilever. The number of ECM stiffness measurements made was operator-determined by the x- and y-step sizes input for a 90µm by 90µm grid. The example shown demonstrates how step sizes of 14 (in the x- and y-directions) result in 196 independent mechanical measurements. b, For regionally-matched correlations between ECM stiffness and mechanosignaling, patient (and xenograft) tissues were immunostained for TNC (green) and pFAK (red). Multiple specific, patient-representative regions (4-10 regions per tumor) were identified (with the x- and y-coordinates noted and images acquired) for further AFM testing in a serial section. For subsequent AFM testing, after patient (and xenograft) tumor sections were secured for testing, the identified testing region was located via x- and y-coordinates using the optical microscope. c, Left: Immunohistochemistry images of patient tumors immunostained for TNC (green) and pFAK (red) with DAPI (blue) with regions for AFM analysis in serial sections denoted by white boxes. Graphs illustrate individual patient correlations between TNC and pFAK. Right: AFM maps of patient tumors. Graphs illustrate individual patient correlations between elastic modulus (stiffness) and pFAK. d, The data underlying (c left) was combined into graphs depicting regional matching of ECM stiffness and pFAK (left) and TNC and pFAK (right) to illustrate not only the correlations between the variables, but also the variability between patients within the cohort (n=3 patient samples/ group, One-way ANOVA with Tukey's comparison P=0.0011). e, The data underlying (c right) was combined into a graph depicting both the patient-specific trends in elastic modulus (stiffness) and the patient variability within the cohort (n=10 patients, 4 regions/patient sample, linear regression analysis yielding p<0.0001 for both plots with R2=0.4812 for pFAK:stiffness data and R2=0.5850 for pFAK:TNC data). Scale Bars 200µm (left panels) and 20µm (right panels), as indicated.

© 2016 Macmillan Publishers Limited, part of Springer Nature. All rights reserved.

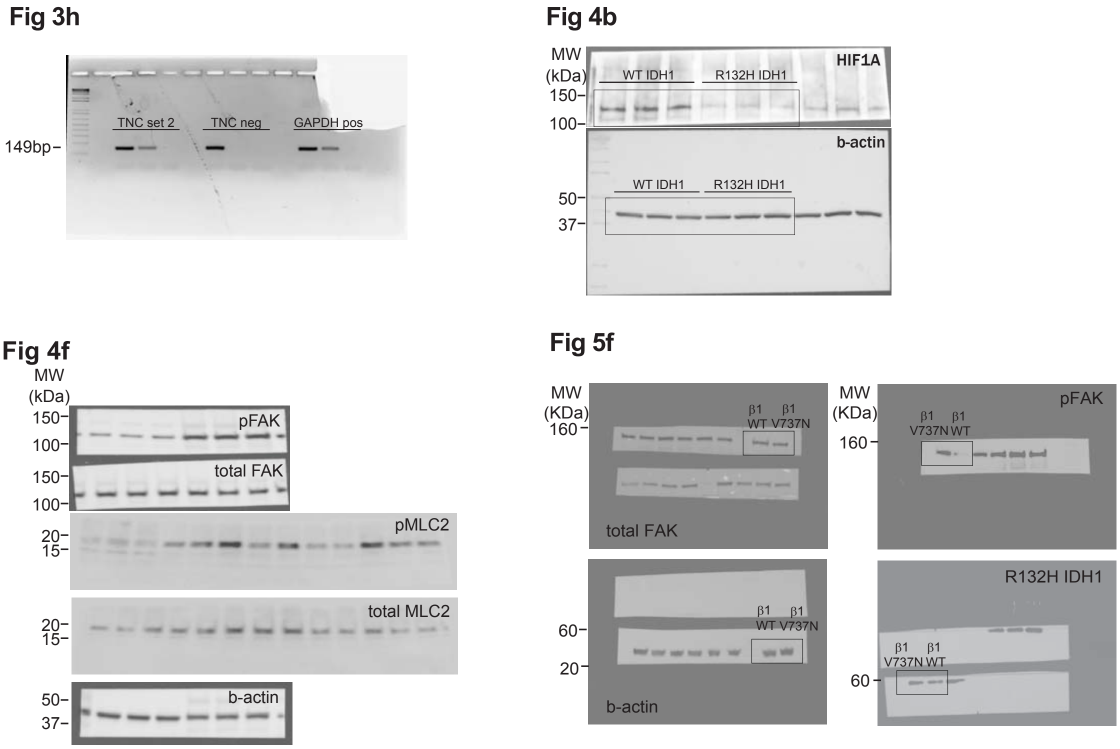

Supplementary Figure 7 Full Scans of Key Blots

© 2016 Macmillan Publishers Limited, part of Springer Nature. All rights reserved.
